# Low concordance of multiple variant-calling pipelines: practical implications for exome and genome sequencing

**DOI:** 10.1186/gm432

**Published:** 2013-03-27

**Authors:** Jason O'Rawe, Tao Jiang, Guangqing Sun, Yiyang Wu, Wei Wang, Jingchu Hu, Paul Bodily, Lifeng Tian, Hakon Hakonarson, W Evan Johnson, Zhi Wei, Kai Wang, Gholson J Lyon

**Affiliations:** 1 Stanley Institute for Cognitive Genomics, Cold Spring Harbor Laboratory, One Bungtown Rd, Cold Spring Harbor, 11724, USA; 2 Stony Brook University, 100 Nicolls Rd, Stony Brook, 11794, USA; 3 BGI-Shenzhen, Shenzhen 518000, China; 4 New Jersey Institute of Technology, Martin Luther King Jr. Blvd, Newark, 07103, USA; 5 Brigham Young University, N University Ave, Provo, 84606, USA; 6 Children's Hospital of Philadelphia, Civic Center Blvd, Philadelphia, 19104, USA; 7 Boston University School of Medicine, E Concord St, Boston, 02118, USA; 8 University of Southern California, 1501 San Pablo Street, Los Angeles, 90089, USA; 9 Utah Foundation for Biomedical Research, E 3300 S, Salt Lake City, 84106, USA

## Abstract

**Background:**

To facilitate the clinical implementation of genomic medicine by next-generation sequencing, it will be critically important to obtain accurate and consistent variant calls on personal genomes. Multiple software tools for variant calling are available, but it is unclear how comparable these tools are or what their relative merits in real-world scenarios might be.

**Methods:**

We sequenced 15 exomes from four families using commercial kits (Illumina HiSeq 2000 platform and Agilent SureSelect version 2 capture kit), with approximately 120X mean coverage. We analyzed the raw data using near-default parameters with five different alignment and variant-calling pipelines (SOAP, BWA-GATK, BWA-SNVer, GNUMAP, and BWA-SAMtools). We additionally sequenced a single whole genome using the sequencing and analysis pipeline from Complete Genomics (CG), with 95% of the exome region being covered by 20 or more reads per base. Finally, we validated 919 single-nucleotide variations (SNVs) and 841 insertions and deletions (indels), including similar fractions of GATK-only, SOAP-only, and shared calls, on the MiSeq platform by amplicon sequencing with approximately 5000X mean coverage.

**Results:**

SNV concordance between five Illumina pipelines across all 15 exomes was 57.4%, while 0.5 to 5.1% of variants were called as unique to each pipeline. Indel concordance was only 26.8% between three indel-calling pipelines, even after left-normalizing and intervalizing genomic coordinates by 20 base pairs. There were 11% of CG variants falling within targeted regions in exome sequencing that were not called by any of the Illumina-based exome analysis pipelines. Based on targeted amplicon sequencing on the MiSeq platform, 97.1%, 60.2%, and 99.1% of the GATK-only, SOAP-only and shared SNVs could be validated, but only 54.0%, 44.6%, and 78.1% of the GATK-only, SOAP-only and shared indels could be validated. Additionally, our analysis of two families (one with four individuals and the other with seven), demonstrated additional accuracy gained in variant discovery by having access to genetic data from a multi-generational family.

**Conclusions:**

Our results suggest that more caution should be exercised in genomic medicine settings when analyzing individual genomes, including interpreting positive and negative findings with scrutiny, especially for indels. We advocate for renewed collection and sequencing of multi-generational families to increase the overall accuracy of whole genomes.

## Background

Recent studies have substantiated the prevalence of rare mutations in the human genome [1,2]. Whole-genome sequencing (WGS) can uncover substantially more genetic variation than traditional single-nucleotide polymorphism (SNP) arrays, thus explaining a larger fraction of human phenotypic diversity [3,4]. This in turn is driving the sequencing of personal genomes aimed at obtaining highly accurate information about each person's genome [5,6].

Given the existence of multiple sequencing platforms and multiple data-analysis pipelines for next-generation sequencing, researchers and clinicians may be under the impression that these methods all work similarly to identify genetic variants from personal genomes. However, one group recently reported that when variants detected in the same sample by the 1000 genomes project (1 KGP) and the Complete Genomics (CG) platform were compared, 19% of the single-nucleotide variants (SNVs) derived were unique to one dataset [7]. This is likely due to differences in technology, data collection, read-alignment methods, and variant-calling algorithms. The group further concluded that 'current research resources and informatics methods do not adequately account for the high level of variation that already exists in the human population, and significant efforts are needed to create resources that can accurately assess personal genomes for health, disease, and prediction of treatment outcomes' [7]. As an illustration of the widely differing methods currently being used, one of the above-referenced papers used Illumina sequencing data processed with the Short Oligonucleotide Analysis Package (SOAP) pipeline [2] whereas the other group used Illumina sequencing data processed with the Genome Analysis Toolkit (GATK) pipeline [1]. Neither group published a comparison of the overlap (concordance or discordance) between pipelines. Other researchers have worked on establishing a rigorous filtering pipeline to optimize SNV calling, reporting that the cumulative application of 12 individual filters resulted in a 290-fold reduction in the error rate [8]. Another group has worked to optimize their own pipeline utilizing, among other things, GATK and SAMtools, although it is not clear if this group compared their results with anything from SOAP [9]. This same group published a comparison of data obtained using sequencing from Illumina and CG, which showed an unexpectedly high level of discordance between the two platforms [10], which has been debated in blog postings [11,12].

Despite these previous studies comparing technical platforms, there have not been many published systematic evaluations of a number of currently used bioinformatics pipelines when generating variant calls from the same set of raw sequence data. Additionally, despite the existence of many variant-calling software tools [13], their concordance using near-default settings has not been thoroughly investigated, making it difficult to assess the relative effects on variant calling of differences in sequencing platforms versus differences in implementations of bioinformatics pipelines. Ideally, researchers and clinicians should have little to no uncertainty about the correct pipeline parameterizations for each sequencing experiment, and hence little variability with respect to their pipeline implementations; however this is rarely, if ever, the case. Indeed, knowledge about the perfect and most appropriate parameterization is often not available or easily obtainable when performing in-depth sequence analysis, and, sometimes the 'correct' parameters may never be precisely characterized due to the complex nature of the experiment. Researchers, clinicians and policy-makers stand to benefit from a greater understanding of the variability introduced by imperfect and non-standardized implementations of the available bioinformatics pipelines.

To address this issue, we carried out a study of 15 exomes and one whole genome from 15 research participants, analyzing the data with a range of different variant-calling pipelines using near-default parameters. Our results have significant implications for analyzing personal genomes from next-generation sequencing experiments.

## Methods

### Ethics approval

The collection and genomic analysis of the DNA were approved by the institutional review board at the University of Utah, and written informed consent was obtained from all study participants. Research was carried out in compliance with the Helsinki Declaration.

### Sample collection

The samples used in our study all came from families of human research participants ascertained in clinics at the University of Utah (see Additional file 1, Figure S1 for pedigrees). Blood samples were collected and genomic DNA extracted using alkaline lysis and ethanol precipitation (Gentra Puregene; Qiagen Corp., Valencia, CA USA). DNA was quality-checked on agarose gels and quantified using a microvolume spectrophotometer (NanoDrop 2000; Thermo Fisher Scientific Inc., West Palm Beach, FL, USA).

### Whole-genome sequencing and analysis with Complete Genomics

After quality control to ensure lack of genomic degradation, we sent DNA samples (10 ug) to Complete Genomics (CG) (Mountain View, CA, USA) for sequencing. The whole-genome DNA was sequenced using nanoarray-based short-read sequencing by ligation technology [14], including an adaptation of the pairwise end-sequencing strategy [15]. Reads were mapped to the Genome Reference Consortium assembly GRCh37. Owing to the proprietary data formats, all the sequencing data quality control, alignment, and variant calling were performed by CG as part of their sequencing service, using their version 2.0 pipeline [16].

### Exome capture and sequencing with Illumina HiSeq2000

Exome capture for all 15 samples was carried out using a commercially available in-solution method (SureSelect Human All Exon v2; Agilent Technologies Inc., Wilmington, DE, USA), following the manufacturer's guidelines. This method is designed to target all human exons, regions totaling approximately 44 Mb, covering 98.2% of the Consensus Coding Sequence (CCDS) database. For the capture, a DNA-shearing instrument (focused-ultrasonicator; Covaris Inc., Woburn, MA, USA) was used to randomly fragment the pure and high molecular weight genomic DNA samples (experiments carried out by BGI-Shenzhen, Shenzhen, China), resulting in DNA fragments with a base-pair peak of 150 to 200 bp. Adaptors were then ligated to both ends of the resulting fragments. The adaptor-ligated templates were purified by magnetic beads (Agencourt AMPure SPRI; Beckman Coulter Inc., Brea, CA, USA), and fragments with an insert size of approximately 250 bp were excised. Extracted DNA was amplified by ligation-mediated (LM)-PCR, purified, and hybridized (SureSelect Library; Agilent Technologies) for enrichment. Hybridized fragments bound to the strepavidin beads, whereas the unbound non-hybridized fragments were washed out after 24 hours of hybridization. Captured LM-PCR products were analyzed using a microfluidics-based platform (2100 Bioanalyzer; Agilent Technologies) to estimate the magnitude of the enrichment. Paired-end sequencing was performed using a sequencing platform (HiSeq2000; Illumina Inc., San Diego, CA, USA) with average read lengths of 90 bp. Raw image files were processed (Pipeline version 1.6; Illumina Inc.) for base-calling, using the default parameters. FASTQ files were produced from the pipeline for downstream sequence data analysis. A gender check was compatible with the known genders of the collected human participants.

### SNP arrays

DNA samples were genotyped on the SNP arrays (Human610-Quad, version 1; Illumina Inc.) with approximately 610,000 markers (including approximately 20,000 non-polymorphic markers) at the Center for Applied Genomics (Children's Hospital of Philadelphia, Philadelphia, PA USA). Total genomic DNA extracted from whole blood was used in the experiments. Standard data-normalization procedures and canonical genotype-clustering files provided by Illumina were used to process the genotyping signals. Concordance between SNPs from the arrays and SNPs from exome sequencing was determined by calculating the percentage of variants from exome sequencing and comparing this with the same genotype derived from the SNP arrays.

### Alignment and variant calling

#### BWA-GATK variant calling

Burrows-Wheeler aligner (BWA; version 0.5.9 [17]) was used to align the sequencing reads, with default parameters, to the human reference genome sequence GRCh37. Alignments were converted from sequence alignment map (SAM) format to sorted, indexed binary alignment map (BAM) files (SAMtools version 0.1.18; http://sourceforge.net). The Picard tool was used to remove duplicate reads. GATK software tools (version 1.5; http://www.broadinstitute.org) were used for improvement of alignments and genotype calling and refining with recommended parameters [18]. BAM files were re-aligned with the GATK IndelRealigner, and base quality scores were re-calibrated by the GATK base quality recalibration tool. Genotypes were called by the GATK UnifiedGenotyper, and the GATK VariantRecalibrator tool was used to score variant calls by a machine-learning algorithm and to identify a set of high-quality SNPs using the Variant Quality Score Recalibration (VQSR) procedure. GATK was used to filter high-quality insertions and deletions (indels) by hard criteria, 'QD < 2.0, ReadPosRankSum < -20.0 FS > 200.0'. Finally, we removed SNVs and indels located outside of regions targeted by exome capture. To increase sensitivity, only those indels with depth (DP) of 10 or more, and with more than 4 reads supporting the indel events were included in the final high-confidence indel set. At a later date, one exome was processed with newer versions of the GATK v2.3-9 UnifiedGenotyper and GATK v2.3-9 HaplotypeCaller modules.

#### BWA-SAMtools genotype calling

Using the above BAM files, we used SAMtools (version 0.1.18) to generate genotype calls [19]. The 'mpileup' command in SAMtools was used to identify SNPs and indels, and we removed variants with DP coverage less than 10, and variants located outside of exome-capture regions.

#### SOAP pipeline

Adaptor and low-quality sequences were removed before mapping. Sequence reads identified from each individual were then aligned to human reference genome GRCh37 using SOAPaligner (version 2.21 [20]) with a maximum of five mismatches. Duplicate reads were removed. Consensus genotypes in target regions were called by SOAPsnp (version 1.03) [21] with recommended parameters. SNV results were filtered (Phred-like SNV quality ≥ 20, overall depth 8 to 500, copy number estimate < 2, and distance between two adjacent SNVs ≥ 5). For a heterozygous SNV, the quality of the minor allele was required to be at least 20, depth of coverage for the minor allele at least 4, and the ratio of major allele to minor allele less than 5. For indel calling, SOAPindel was used, which adopts local assembly based on an extended de Bruijn graph [22]. For SOAPindel, the aligner BWA was used to align the reads to the human reference sequence with default parameters. Initially, putative indels were assumed to be located near the unmapped reads whose mates mapped to the reference genome. SOAPindel then executed a local assembly (k-mer = 25) on the clusters of unmapped reads. Clusters with coverage of less than 5 were not used. The assembly results were aligned to the reference in order to find the potential indels. To distinguish true-positive and false-positive indels, SOAPindel generates Phred quality scores, which take into consideration the depth of coverage, indel size, number of neighboring variants, distance to the edge of the contig, and position of the second different base pair. Only those indels with a quality score of 10 or higher were retained in the final indel call set.

#### GNUMAP pipeline

Diploid and monoploid SNVs for each individual were called using the GNUMAP pipeline (version 3.1.0 [23]). GNUMAP-SNP utilizes a novel probabilistic pair-hidden Markov model, which accounts for uncertainty in the read calls as well as read mapping in an unbiased fashion. Raw reads were initially aligned to the full genome using an alignment score of 260 or greater, which for this dataset allowed for only one SNV per read. A k-mer size of 12 and a jump size of 10 were also used. Only SNVs within exome regions with a *P *< 0.001 were reported. The GNUMAP pipeline cannot currently call indels.

#### BWA-SNVer pipeline

BWA [17] was used to align the sequencing reads to GRCh37 with default parameters. Duplicate reads were removed by Picard, and SNVer (version 0.2.1) was then used for detecting SNVs in each sample [24]. Similar to GATK [18], only the mapped short reads with mapping quality of greater than 20 were considered, and only bases with base quality greater than 17 counted. SNVer estimated the empirical error rate for those selected reads in making variant calls. We set the number of haploids to 2 for analysis of individual samples, and set the variant allele frequency threshold of greater than 0 for detecting both rare and common SNVs. SNVer provides multiplicity control, and we performed Bonferroni correction and controlled the family-wise error rate at the 0.05 level to report identified SNVs. Indels cannot currently be called by the BWA-SNVer pipeline.

### Post-variant calling analyses

Post-variant-calling analyses were performed using Golden Helix SVS (version 7.6.10 [25], ANNOVAR [26], the R suite of statistical programming tools http://www.r-project.org, and custom Perl scripts.

### MiSeq sequencing for validation

Validation variants were randomly selected from sets of particularly controversial variants, indels and SNVs unique to GATK, indels and SNVs unique to SOAP, and variants (both SNVs and indels) shared by these two pipelines. PCR primers were designed using the software program Primer 3 http://sourceforge.net, to produce amplicons (ranging in size from 100 to 200 bp) containing variants of interest in approximately the center of the amplicon. Primers were obtained in 96-well plate format, 10 μmol/L dilution each (Sigma-Aldrich, St Louis, MO, USA). All primers were first tested for PCR efficiency using a HAPMAP DNA sample (Catalog ID NA12864l Coriell Institute for Medical Research, Camden, NJ, USA) and DNA polymerase (LongAmp^® ^Taq DNA Polymerase; New England Biolabs, Beverly, MA, USA). k8101-49685 genomic DNA was used as template for the validation experiment. After quality-control steps using agarose gel, the product was purified (ExoSAP-IT^® ^reagentsl Affymetrix Inc., Santa Clara, CA, USA) and pooled. Final PCR products were quantified (Qubit^® ^dsDNA BR Assay Kitl Invitrogen Corp., Carlsbad, CA, USA), then library construction for the sequencer platform (MiSeq Personal Sequencer; Illumina Inc.) was performed. Finally, before being loaded onto the MiSeq machine, the quality and quantity of the sample was verified using the Bioanalyzer (Agilent Technologies) and quantitative PCR (Kapa Biosystems Inc., Woburn, MA, USA).

### Accessing data

All of the data have been submitted to the Sequence Read Archive under project accession SRP019719, corresponding to the 15 exomes and the single whole genome analyzed during the course of our study (see Additional file 2).

## Results

### Data production summary

Fifteen DNA samples from four different families (see Additional file 1, **Figure S1**) were prepared by exon capture (Agilent 44 MB SureSelect protocol; Agilent Technologies), followed by sequencing on (HiSeq2000; Illumina Inc.). On average, we obtained sequence coverage of approximately 120X (range, 100 to 154X) on targeted regions for these 15 samples. For all samples, sequence reads covered more than 80% of the targeted region with a depth of greater than 20 reads per base (see Additional file 1, Figure S2; for data production statistics, see Additional file 3). Five different pipelines were used for read alignment and variant calling (SNVs and indels when possible) (Table 1). In addition, one whole genome was sequenced and analyzed by CG with 95% of the exome region covered by 20 reads or more per base, resulting in greater than 88% of the genome covered with a depth of greater than 20 reads per base. Variant calls were generated by CG with their in-house analysis pipeline (version 2.0).

**Table 1:** A descriptive summary of the variant calling pipelines included in the comparative analyses.

Pipeline name	Alignment method	Variant-calling module	Description of variant-calling algorithm
SOAP	SOAPaligner version 2.21/BWA version 0.5.9	SOAPsnp version 1.03/SOAPindel version 2.01	SOAP uses a method based on Bayes' theorem to call consensus genotype by carefully considering the data quality, alignment, and recurring experimental errors [22].
GATK version 1.5	BWA version 0.5.9	UnifiedGenotyper version 1.5	GATK employs a general Bayesian framework to distinguish and call variants. Error correction models are guided by expected characteristics of human variation to further refine variant calls [19].
SNVer version 0.2.1	BWA version 0.5.9	SNVerIndiversion idual version 0.2.1	SNVer uses a more general frequentist framework, and formulates variant calling as a hypothesis-testing problem [25].
GNUMAP version 3.1.0	GNUMAP version 3.1.0	GNUMAP version 3.1.0	GNUMAP incorporates the base uncertainty of the reads into mapping analysis using a probabilistic Needleman-Wunsch algorithm [24].
SAMtools version 0.1.18	BWA version 0.5.9	mpileup version 0.1.18	SAMtools [20] calls variants by generating a consensus sequence using the MAQ model framework, which uses a general Bayesian framework for picking the base that maximizes the posterior probability with the highest Phred quality score.

### SNV analysis

#### Concordance with SNP genotyping arrays

Sensitivity and specificity for detecting common SNPs was calculated for each Illumina variant-calling pipeline for four samples that were genotyped with the Illumina Human610-Quad version 1 SNP array (see Additional file 1, Table S1). We caution that this analysis was restricted to a set of common SNPs targeted by the SNP array, and that these tend to be within regions containing little to no repeated sequences and without extreme GC contents. Therefore, although widely used in published literature, concordance with SNP arrays does not adequately measure real-world performance on all variants in personal genomes. With this major caveat in mind, performance for each pipeline was measured by treating the Illumina Human610-Quad version 1 SNP arrays as a true-positive reference, and comparing the exome-capture sequencing results with this reference set. The average specificity for each of the five Illumina pipelines was generally high, ranging from 99.59% to 99.87% (Table 2), consistent with the fact that each of these pipelines have been optimized to minimize false negatives for known common SNPs. The average sensitivity ranged among the five pipelines from 86.6% (with GNUMAP) to 95.3% (with GATK1.5). Sensitivity decreased when the variant set was iteratively restricted to the intersection between two or more variant-calling pipelines, whereas specificity naturally shows the opposite trend of increasing values under the same series of intersections (Table 2).

**Table 2:** Quality evaluation of variant detection using different variant-calling pipelines.

	Sensitivity		Specificity	
	Mean*	SD	Mean*	SD
SOAPsnp	94.68	2.26	99.79	0.03
GATK1.5	95.34	1.16	99.72	0.08
SNVer	92.33	4.40	99.78	0.04
GNUMAP	86.60	3.23	99.64	0.06
SAMtools	94.47	4.22	99.59	0.16
Any pipeline	97.67	1.20	99.62	0.11
≥ 2 pipelines*	96.64	2.28	99.69	0.07
≥ 3 pipelines*	95.62	3.13	99.73	0.05
≥ 4 pipelines*	92.60	3.40	99.82	0.04
5 pipelines*	80.58	5.26	99.87	0.01

*Intersection of variants contained in the number of pipelines specified.

Sensitivity and specificity was calculated for each pipeline by comparing Illumina Human610-Quad version 1 SNP arrays with exome-capture sequencing results, based on the four samples whose genotyping data was available.

#### Evaluation of performance by inheritance analysis

To explore the validity of SNVs called by each Illumina pipeline, we performed an inheritance analysis for two families contained within the 15 sequenced exomes. Previous calculations have estimated the average expected number of *de novo *non-synonymous coding mutations per individual exome to be approximately 1 to 2 [27-30]. However, we found that the number of putative *de novo *mutations per child per exome was much higher if only the parents of the child were used to filter out inherited mutations. Adding an additional familial generation to the filtering process, in our case a grandparent, significantly reduced the number of putative *de novo *variants to a value comparable with that of the previously reported value of expected *de novo *non-synonymous mutations. In addition, significant variation was seen in the number of putative *de novo *mutations between the two families (Table 3), consistent with previous findings [31].

**Table 3:** *De novo *single-nucleotide variants (SNVs) were detected in two families contained within the 15 study exomes.

	Number of putative *de novo *coding non-synonymous or nonsense SNVs detected	
**Family 1**	Without using the grandparents as a filter	Using the grandparents as a filter
Child A	241	1
Child B	211	0
Child C	102	6
Child D	242	3
**Family 2**		
Child A	49	NA^a^
Child B	41	NA^a^

^a^N/A, no grandparent available.

Family 1 had a grandparent available for filtering purposes, whereas family 2 did not. To minimize false positives in the pool of SNVs associated with each child, only highly concordant SNVs were used (SNVs detected by all five pipelines). To construct a comprehensive set of SNVs for each parent, and hence increase filtering accuracy, false negatives for parent SNVs were reduced by taking the union of all SNV calls from all five pipelines.

#### Variant-calling pipeline concordance

**Figure 1:**
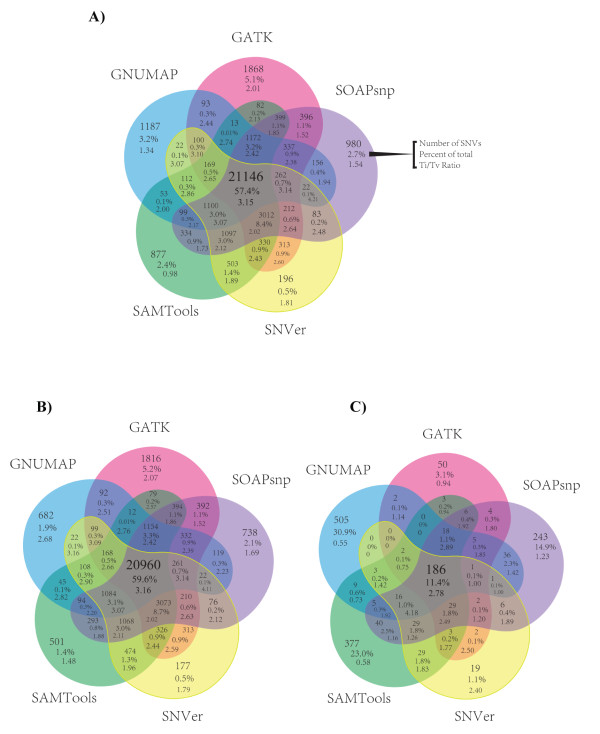
**Mean single-nucleotide variants (SNV) concordance over 15 exomes between five alignment and variant-calling pipelines**. The alignment method used, followed by the SNV variant calling algorithm is annotated here in shorthand: BWA-GATK, SOAP-Align-SOAPsnp, BWA-SNVer, BWA-SAMtools, and GNUMAP-GNUMAP. **(A) **Mean SNV concordance between each pipeline was determined by matching the genomic coordinate as well as the base-pair change and zygosity for each detected SNV. **(B) **The same analysis as in (A) but filtered to include only SNVs already found in dbSNP135. **(C) **The same analysis as in (A), but filtered to include novel SNVs (that is, SNVs not found in dbSNP135).

SNV concordance between all 5 Illumina pipelines across all 15 exomes was 57.4% on average, and Ti/Tv ratios showed a generally increasing trend for sets of variants intersected by an increasing number of variant-calling pipelines (Figure 1). We found that for novel SNVs (those not found in dbSNP135) the overall concordance (11.4%) was much lower than the overall concordance between known SNVs (59.6%) (Figure 1). In a previous paper, we validated with Sanger sequencing or Sequenom genotyping 17 SNVs found in 3 of the current pilot samples [32]. Of these 17 validated SNVs, 16 were detected by all 5 pipelines, and the remaining variant was called by 4 of the 5 pipelines. Additional validation analyses are presented later in this paper.

A more detailed analysis of SNVs of one sample (k8101-49685) revealed that the exome variant calls had moderate to high depth of coverage (see Additional file 1, Figure S3). The range of read depths along with read-depth uniformity of variant calls varied between pipelines (see Additional file 1, Figure S3). Overall concordance between all five pipelines for sample k8101-49685 was 57.5%; however, sub-setting variants called by Illumina pipelines using increasingly stringent read-depth thresholds did not increase SNV concordance (see Additional file 1, Figure S4), and overall concordance was lowest when read-depth threshold values were at their highest (32.7% concordance when depth was required to be greater than 30 supporting reads).

#### Sequencing platform concordance

**Figure 2:**
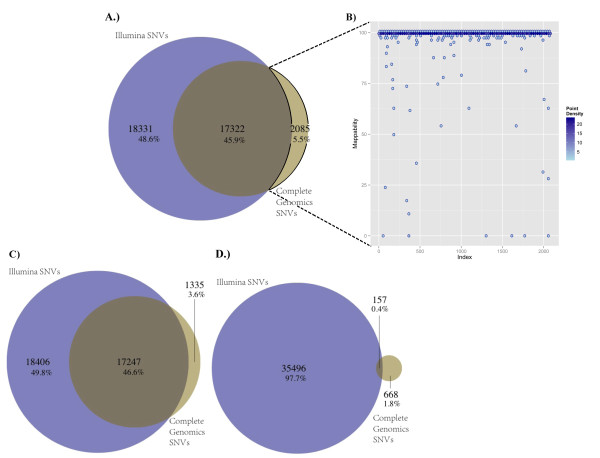
**Single-nucleotide variant (SNV) concordance, between two sequencing pipelines (Illumina and Complete Genomics (CG)) for a single exome, k8101-49685**. For the Illumina sequencing, exons were captured using the Agilent SureSelect version 2 panel of capture probes. CG SNVs consisted of a subset of all SNVs called by CG that fell within the Agilent SureSelect version 2 exons. Concordance was determined by matching the genomic coordinates, base-pair composition, and zygosity status for each detected SNVs. Illumina SNVs consisted of all SNVs (the union) called by the five variant-calling pipelines GATK, SAMtools, SOAPsnp, SNVer, and GNUMAP, which increased the false positives but decreased the false negatives. Concordance was measured between Illumina SNVs and **(A) **all CG SNVs, **(C) **only high-quality (VQHIGH) CG SNVs, and **(D) **only low quality (VQLOW) CG SNVs. **(B) **Genome mappability analyses were performed on 2,085 discordant SNVs, which were found by the CG pipeline and not found by any of the five Illumina data pipelines.

For sample k8101-49685, we selected variants generated by the CG pipeline that fell within the exon-capture regions of the Agilent SureSelect version 2 capture kit. We found that of the 21,050 SNVs identified by CG and located within the UCSC refGene regions, 19,407 (92%) were also within regions targeted for capture by the Agilent SureSelect version 2 kit. Of these, 2,085 (11%) were not called by any of the Illumina-based exome-analysis pipelines, despite computed high mappability scores for these variants (Figure 2) [33]. Of these 2,085 SNVs uniquely called by CG, an average of 558 had no sequence coverage as mapped by any of the Illumina-based exome-analysis pipelines. The Illumina exome read-depth for the remaining 1,527 CG-unique SNVs was calculated, and the majority of these SNVs were found to be in regions of very low Illumina sequence coverage (< 20 reads) in the exome datasets (see Additional file 1, Figure S5).

We found that 89.3% of CG SNVs (17,322 of 19,407) were contained within the union of all five Illumina pipelines (35,653 putative SNVs), whereas 18,331 of these 35,653 putative Illumina SNVs were not called by CG, suggesting a high false positive rate in the union of the Illumina calls and/or conversely a high false-negative rate in the CG calls (Figure 2). Overall concordance displayed marginal increases when VQLOW SNVs (low-quality CG variants) were removed from the pool of CG SNVs (Figure 2). Overall concordance remained stable as the depth of coverage threshold value associated with Illumina data calls increased (see Additional file 1, Figure S6A).

When only highly concordant Illumina SNVs (SNVs called by all five Illumina pipelines) were compared with the CG SNVs, only 64.4% (12507) of CG SNVs were contained within the concordant Illumina set, suggesting a high false-negative rate in this highly concordant Illumina set. Overall agreement decreased as the depth of coverage threshold value for Illumina calls increased, consistent with an increasing false-negative rate (see Additional file 1, Figure S6B).

#### Cross-platform comparison of unique-to-pipeline SNVs

SNVs from sample k8101-49685 that were uniquely detected by only one of the five Illumina variant-calling pipelines were compared with SNVs called by CG (see Additional file 1, Figure S7). Of the SNVs uniquely called by GATK, 809 of 1671 (48%) were concordant with CG data. The concordance was much lower for the other four pipelines, 49 of 1,102 SNVs (4%) for GNUMAP, 45 of 886 (5%) for SAMtools, 29 of 226 (12%) for SNVer, and 24 of 908 (3%) for SOAPsnp. Concordance improved for SNVs that were called by more than a single Illumina data pipeline, and the concordance was the highest for variants found by all five Illumina pipelines (see Additional file 1, Figure S7).

For variants that were novel as well as unique to a single Illumina pipeline, concordance with CG data was low (see Additional file 1, Figure S7). For GATK, 25% (13 of 51) of novel and unique-to-pipeline SNVs were concordant with CG data; for GNUMAP and SOAPsnp, no novel and unique-to-pipeline SNVs were concordant (0 of 470 and 0 of 229 respectively); for SAMtools, 0.2% (1 of 418) of novel and unique-to-pipeline SNVs were concordant; and for SNVer, 6% (1 of 18) of novel and unique-to-pipeline SNVs were concordant. Concordance rates of novel and unique-to-pipeline SNVs increased for variants called by an increasing number of pipelines (see Additional file 1, Figure S7).

### Indel analysis

#### Variant-calling pipeline concordance

**Figure 3:**
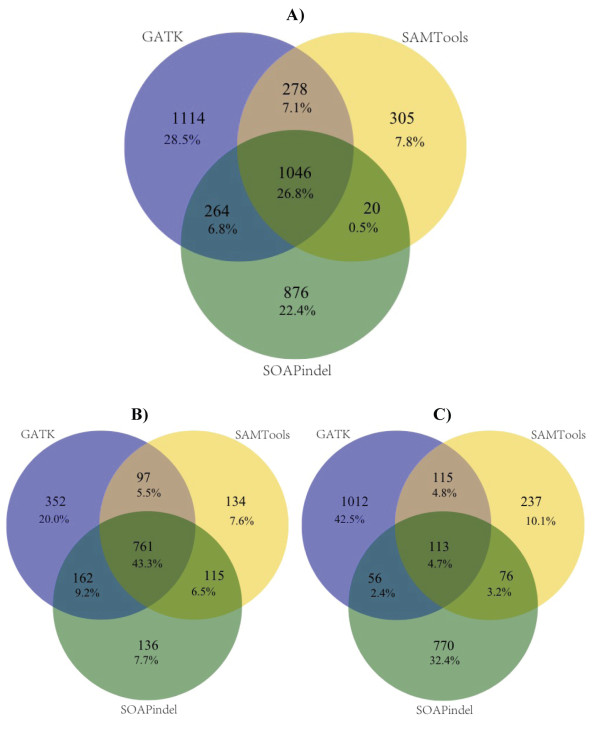
**Mean indel concordance over 15 exomes between 3 indel-calling pipelines: GATK, SOAPindel, and SAMtools**. Mean concordance was measured between **(A) **all indels, **(B) **known indels (indels found in dbSNP135), and **(C) **unknown indels (indels not found in dbSNP135). Indels were left normalized and intervalized to a range of 20 genomic coordinates (10 coordinates on each side of the normalized position) to allow for a reasonably standardized indel metric for comparison. To determine whether or not indels were matching, the genomic coordinates as well as the base length and composition of each indel were considered.

For indel calls, initial agreement between SOAPindel, SAMtools and GATK was very low at 3.0% (see Additional file 1, Figure S8). Indel coordinates were subsequently left-normalized and intervalized using a total range of 20 genomic coordinates (10 bp in each direction of their genomic coordinates). We found that increasing the intervalized indel range to as much as 60 genomic coordinates only marginally differed from having 20, so we chose to use 20 as a reasonable and conservative range for intervalizing indels. This method increased the overall concordance to 26.8% between the three indel-calling pipelines (Figure 3). For novel indels, the concordance (4.7%) was much lower than the overall concordance among known indels (43.3%). In an earlier paper, we previously validated with Sanger sequencing three indels found in three of the current pilot samples [32]. These three validated indels were detected by all three indel-calling pipelines.

#### Sequencing platform concordance

**Figure 4:**
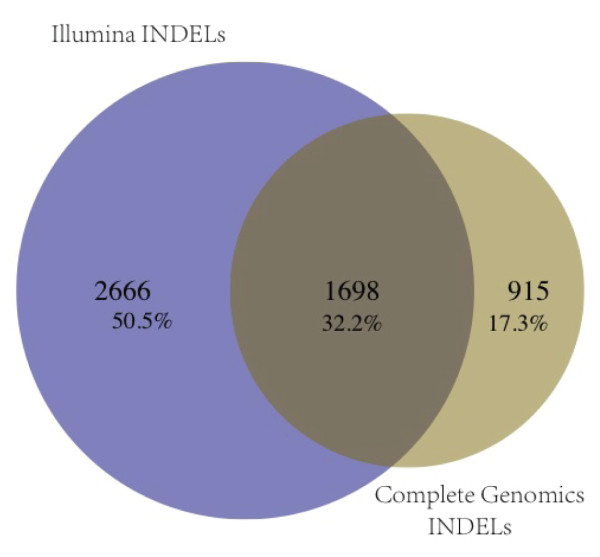
**Indel concordance for a single exome, k8101-49685, between two sequencing pipelines: Illumina and Complete Genomics (CG)**. Illumina indels consist of a union of all indels called by each of the three indel-calling pipelines GATK, SOAPindel, and SAMtools. CG indels consisted of a subset of indels called by CG that fell within the Agilent SureSelect version 2 exons. Both Illumina and CG indels were left normalized and intervalized to a range of 20 genomic coordinates (10 coordinates on each side of the normalized position). To determine whether or not indels were matching, the genomic coordinates as well as the base length and composition of each indel were considered.

Indels falling within the range of the Agilent SureSelect version 2 exons were excised from the whole genome of sample k8101-49685 sequenced and analyzed by CG. Indels were again left-normalized and intervalized using a total range of 20 genomic coordinates, 10 in either direction. The normalized and intervalized CG indel calls were compared with all normalized and intervalized indels detected by the three Illumina data pipelines, and 32% were in agreement (Figure 4).

##### Cross-platform comparison of unique-to-pipeline indels

For the three Illumina data indel-calling pipelines, unique-to-pipeline indels were compared with indels discovered by CG (see Additional file 1, Figure S9). Concordance with CG indels was relatively low for all three pipelines with unique-to-GATK indels, showing a concordance of 24% with CG indels (324 of 1366), unique-to-SAMtools showing 29% concordance with CG indels (142 of 498) and unique-to-SOAPindel having 12% of called indels being concordant with CG indels (147 of 1246). Concordance rates improved for variants that were called by two Illumina data pipelines and further improved for variants called by all three of the indel-calling pipelines, 63% (1241 of 1986) and 90% (963 of 1069), respectively.

Novel (to dbSNP135) and unique-to-pipeline indels were also compared to CG indel calls, and the concordance rates for each pipeline were similar to those of the unique-to-pipeline only variants (see Additional file 1, Figure S9). Novel and unique-to-GATK indels had 24% of its indels concordant with CG indels (299 of 1236), novel and unique-to-SAMtools had 28% of its indels concordant with CG indels (96 of 343) and novel and unique-to-SOAPindel had 5% of its indels concordant with CG indels (53 of 1056). Novel indels that were called by two Illumina data pipelines displayed an increased concordance rate, 54% (229 of 423). Variants called by all three of the indel-calling pipelines showed the highest concordance rate for novel, unique-to-pipeline indels, 84% (103 of 122).

### MiSeq validation of pooled PCR amplicons

To validate variants called by the two more widely used pipelines (SOAP and GATK), we used the orthogonal approach of PCR amplification of genomic DNA regions containing selected SNVs and indels, followed by pooled MiSeq sequencing. The PCR amplification (instead of exon capture), longer read lengths on the MiSeq platform, and the much higher depth of coverage provided a strong method of validation for SNVs and indels. A total of 1,140 SNVs found in sample k8101-49685 were selected for MiSeq validation; 760 of these SNVs were randomly selected from the set of SNVs that were unique to the GATK version 1.5 and SOAPsnp version 1.03 pipelines, 380 SNVs from each pipeline respectively. An additional 380 SNVs were randomly selected from the set of variants that were in agreement between GATK and SOAPsnp. After some analysis of the quality of the MiSeq data using FASTX, the MiSeq paired-end read data (version 2 sequencing kit, 250 × 250 bp reads) was trimmed to 150 bp and then aligned with BWA version 0.6.2 to the human reference genome sequence GRCh37, and variants were called with GATK UnifiedGenotyper version 2.3-9.

**Figure 5:**
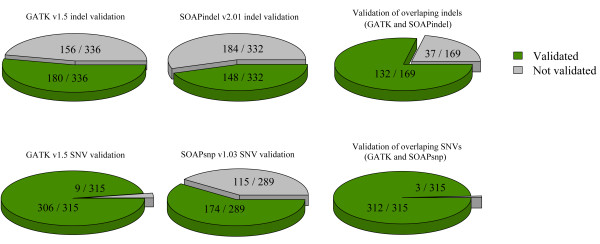
**MiSeq validation experiment on a subset of Illumina-data calls**. A total of 1,140 SNVs from sample k8101-49685 were randomly sampled for MiSeq validation, with 380 sampled from the set of unique-to-GATK SNVs, 380 sampled from the set of unique-to-SOAPsnp SNVs, and 380 sampled from the set that were overlapping between these two pipelines. There were 919 (81.0%) of these variants that were successfully amplified and sequenced. BWA version 0.6.2 and GATK version 2.3-9 were used to process the sequencing data for variant-calling. Additionally, 960 indels from sample k8101-49685 were randomly selected for validation. Of these, 386 were randomly selected from the unique-to-GATK indel set, 387 were randomly selected from the unique-to-SOAPindel set, and 187 were randomly selected from the set of indels overlapping between the two (SOAPindel and GATK). There were 841 (83.5%)of these indels that were successfully amplified and sequenced. BWA version 0.6.2 and GATK version 2.3-9 were used to determine the number of variants that were also successfully validated across these sets.

Of the 1,140 SNVs targeted for MiSeq validation, 919 (81.0%) were successfully amplified and sequenced, with an average read depth of 5,392. Validation rates for unique-to-GATK SNVs were high, with 306 of 315 (97.1%,) being successfully validated. For unique-to-SOAPsnp, 174 of 289 SNVs (60.2%) were validated. SNVs that were called by both GATK and SOAP were validated in 312 of 315 instances (99.1%) (Figure 5).

For indels found in sample k8101-49685, 960 were randomly selected for validation. Of these, 386 were randomly selected from the unique-to-GATK indel set, 387 were randomly selected from the unique-to-SOAPindel set, and 187 were randomly selected from set of indels overlapping between the two (SOAPindel and GATK). Of the 960 indels that were targeted for sequencing, 841 (83.5%) were successfully amplified and sequenced, with an average coverage of 4,866.

Unique-to-GATK indels had a validation rate of 180 of 336 (54.0%), being validated. The validation rate for unique-to-SOAPindel was found to be 44.6%, with 148 of 332 validating. For indels that were called by both SOAPindel and GATK, 132 of 169 (78.1%) were successfully validated (Figure 5).

### GATK v2.3-9 and the new HaplotyperCaller

Newer implementations of SNV and indel-calling pipelines continually advance the field of variant discovery and analysis by increasing the accuracy by which variants can be reliably called. Here, we show an example of the differences between previous versions of GATK with respects to SNV calls and indel calls on the same sample, k8101-49685. The vast majority of SNV calls made by both the GATK UnifiedGenotyper version 2.3-9 and the GATK HaplotypeCaller version 2.3-9 modules overlapped with the SNV calls made by the GATK UnifiedGenotyper version 1.5, showing an overall concordance of 91.0% (27,150 of 29,912) and 87.0% (26,751 of 30779) respectively. However, for indel calls, the picture was quite different, with the GATK UnifiedGenotyper version 2.3-9 and GATK HaplotypeCaller version 2.3-9 modules showing an overall concordance with the GATK UnifiedGenotyper version 1.5 calls of 54.7% (1,688 of 3,085) and 54.6% (1,858 of 3,404) respectively (see Additional file 1, Figure S10).

## Discussion

Significant advances have been made in detecting genomic variation in 'next-generation' sequencing data, despite considerable sources of uncertainty [13]. However, we have found that there still exists significant discrepancy between the overall variant sets called by five different variant-calling pipelines applied to the same raw sequencing data using near-default parameters, along with differences noted between two next-generation sequencing (NGS) platforms. There are, of course, relatively large regions of overlap between all pipelines even when highly specialized parameterizations are not used. This suggests that there exists a 'region' of variants that can be called robustly by many different pipelines regardless of meticulous parameterization. The field has naturally focused on this robust set of calls, but we wish to highlight here the considerable degree of discordance as well as the high false-negative rates.

### A discussion about variant quality and the case for multiple methods for sequence analysis in personal genomes

For the five variant-calling pipelines included in our study, a large number of calls (both SNV and indel) are shared among them in each exome, 21,146 on average for SNVs. Although all five pipelines converge on a relatively large number of SNVs, this still represents less than 60% of the total SNV call set determined by all five pipelines, and hence there still exists a considerable degree of variation between pipelines used, with near-default parameterization on the same raw sequencing data. This disagreement is likely to be the result of many factors including alignment methods, post-alignment data processing, parameterization efficacy of alignment and variant-calling algorithms, and the underlying models utilized by the variant-calling algorithm(s).

SAMtools, SOAPsnp, and GATK use similar Bayesian methods to compute the posterior probability of the true genotype [19,34-37], but they differ in the prior probabilities used. For example, SAMtools (MAQ) sets the prior probability for a heterozygous SNV at 0.001 for novel variants, and 0.2 for known SNVs. SOAPsnp uses a more complex method of assigning prior probabilities by distinguishing the homozygous genotype for the reference allele from the homozygous genotype for the alternative, and distinguishing transition (A↔G, C↔T) mutations from transversion (A/G↔C/T) mutations. GATK is similar to SAMtools but utilizes more advanced pre-processing and post-processing steps, such as local re-alignment around possible indel loci, and quality recalibration of both base quality and variant quality to improve overall variant-call performance. By modeling allele frequency, SNVer uses a frequentist framework for calling variants [24]. SNVer formulates variant calling as a hypothesis-testing problem so that a prior probability is not required, and SNVer could act as a complementary method to Bayesian methods. GNUMAP, which employs a probabilistic Needleman-Wunsch algorithm, might also be considered an orthogonal method, as even its computational framework for sequence alignment is novel [23].

Each variant-calling pipeline detects variants that others do not, and the accuracy of these *dis*cordant variants is expected to be low, but not zero. Indeed, for our comparison of SNVs called by SOAP, GATK, or both, MiSeq validation of unique-to-GATK and unique-to-SOAP variants demonstrated relatively high rates of validation, with 306 of 315 of the randomly selected SNVs from unique-to-GATK and 174 of 289 SNVs from unique-to-SOAPsnp being validated. Indels had lower, but still non-zero, validation rates, with 180 of 336 unique-to-GATK indels and 148 of 332 unique-to-SOAPindel indels being validated. Given that 'unique-to-pipeline' variants exist even in regions of relatively high sequence coverage, it is necessary to develop other approaches for including or excluding these variants from downstream analyses.

In the realm of biomedical research, every variant call is a hypothesis to be tested in light of the overall research design. Missing even a single variant can mean the difference between discovering a disease-contributing mutation or not [38]. For this reason, our data suggest that using a single bioinformatics pipeline for discovering disease-related variation is not always sufficient. A more comprehensive approach can be taken; all variants discovered by multiple variant-calling pipelines, when coupled with appropriate no-calling and quality filtering, could be included in downstream analyses, so as to not miss potentially disease-contributory variants. This is something that we intuitively implemented in a prior project [39], but for which these data now provide empirical support.

One can minimize false positives by increasing stringency filters, but this automatically and correspondingly increases the false-negative rate. The intersection between multiple variant-calling pipelines will minimize the false-positive rate, but we have also shown that each pipeline uniquely identifies some true variants. Hence, clinicians and policy-makers should be aware that propagating forward the intersection of variant calls will result in a high false-negative rate with exome sequencing and WGS, and we discuss below the advantage brought to bear on this issue by studying families. Therefore, processing single sample datasets from one sequencing platform with multiple variant-calling pipelines should not be the long-term solution for generating variant calls with high sensitivity and specificity, and we discuss alternative approaches below.

### The case for indel standardization

Although the focus of most variant-calling software has been on detecting SNVs, it is the case that large-scale structural copy number variants and small indels are known to also be a biologically relevant and prevalent form of genetic variation [40,41]. Indeed, initial indel mapping efforts revealed upwards of 800,000 indels in a diverse population that map to known human genes, some of which can be associated with genetic disease [42,43], while recent estimates from the 1000 Genomes Project [44] suggest a 10:1 ratio of SNVs to indels in individual human genomes. Reliably detecting indels is therefore a crucial component of constructing a comprehensive set of clinically relevant genetic variants.

In contrast to SNVs, few indel-calling tools have been developed, so current knowledge of the existing variation due to indels, as well as the clinical implications of indels, has lagged. In spite of the fact that indel detection is becoming an important aspect of structural-variant analysis [40], indel calling is relatively imprecise and inaccurate. For example, the position of an indel with respect to its reference is, in many cases, ambiguous. An indel can often be represented at any of multiple locations. Krawitz *et al. *[45] designed an indel coordinate comparison metric, the equivalent indel region (eir), for comparing indel calls between pipelines, and GATK provides a tool which attempts to normalize indel position by left-justifying the indel within its multiple possible coordinate representations. Indeed, commonly used databases such as dbSNP have not yet entirely addressed the imprecision of indel-calling pipelines [45] and report only a single position for an indel, which could lead to disparate clinical diagnoses/outcomes between similarly affected individuals. We suggest that a more comprehensive approach should be taken, with all potential positions for each indel expressed and accounted for, so that downstream analysis can take advantage of the known existing ambiguity.

Our data demonstrates large discrepancies between indel-calling pipelines and suggests potentially high numbers of false positives and/or false negatives. Although putative false positives can be tested via modest resequencing efforts, false negatives and 'no-calls' require large-scale, often impractical, resequencing projects to discover them. Because of this limitation, few data exist on false-negative rates across pipelines, and inferring these rates from unique calls between pipelines is likely to be inadequate. Indel frequency, indel size, read length, and read depth are all known to affect the accuracy of indel-calling pipelines, and the performance of different pipelines also depends, in part, on experimental conditions [46]. We show that a relatively simple method can increase comparison accuracy for indels between pipelines and between individuals; left-normalized and intervalized indel calls allow rapid and reasonable comparison of called indels between different indel-calling pipelines, as well as between individuals who have had their genome or exome sequenced. The issues highlighted in our indel comparisons demonstrate the difficulties associated with attaining accurate and standardized indel calls, and our data illustrate the need for robust and ubiquitous indel standardization metrics/methods to allow for objective comparisons across pipelines and across sequencing projects.

### The case for studying large families for discovering disease-related genetic variation

Our analysis of two families, one containing only two generations (parents and children) and the other containing three generations (one grandparent, both parents, and children) demonstrates that the ability to accurately distinguish d*e novo *variants from familial inherited variants may be more strongly limited by high false-negative rates in the parents than by high false-positive rates in the children. This can be significantly improved by having sequence data from one or more grandparents or other relatives. This finding is particularly salient for single-generation *de novo *studies, which attempt to characterize novel variants that are associated with genetic disease observed in the children of healthy parents [47-51]. While such 'no-call' or false-negative errors in parents can be ameliorated somewhat with higher sequencing depth and/or more comprehensive variant-calling strategies, larger and more comprehensive pedigrees provide a powerful, complementary source for discovering and studying human genetic variation. Although most studies utilizing NGS data to date have focused on 'quads' or 'trios' [47-51], studying large and/or consanguineous families can maximize the utility of filtering strategies and statistical approaches for identifying disease-contributory variants in genetic disease [52].

### The case for different platforms for a more comprehensive "exome"

The relative merits of WGS are expanding as both the cost of the technique decreases and as more scientists and clinicians use the technique in an increasing number of studies/analyses [5,30,53,54]. We have shown that WGS with the version 2.0 CG pipeline delivers SNVs and indels not discovered by the Agilent exon capture and Illumina sequencing, and that these variants have a very low number of reads (< 20) at those positions in the Agilent exomes, arguing that the capture of those regions was not efficient. Conversely, there were a significant number of variants in our data that were unanimously called by the five Illumina variant-calling pipelines but not by the CG WGS. Exon capture and sequencing at high depth with one platform can yield a much higher depth of coverage in most exonic regions, whereas WGS offers a more uniform and comprehensive coverage that appears to cover regions missed by exon capture and sequencing. To attain a truly comprehensive set of exonic variants, WGS on one platform could be combined with exon capture and sequencing on a different platform. This combination of the depth of exonic sequencing provided by exon capture with the breadth of coverage of WGS on a different sequencing platform, alongside the use of multiple variant-calling pipelines, provides a powerful means to maximize sensitivity and specificity for any one personal genome. However, as costs for sequencing are reduced, we anticipate that sequencing whole genomes on two or more platforms may become a feasible option for similarly maximizing accuracy.

### The current state of variant discovery

Many of the most recent advancements that have been made in variant discovery and sequencing analysis are those related to indel discovery and analysis. Indeed, newer versions of GATK have improved upon the false-negative and false-positive rates of their indel calls in both UnifiedGenotyper and with the newer HaplotypeCaller. By leveraging local *de novo *assembly, similarly to the SOAPindel pipeline used in this study, the new HaplotypeCaller from GATK potentially greatly improves upon its indel-calling accuracy. The more distinct differences observed between GATK indel calls by different versions reflects the fact that indel discovery is in the earlier stages of development, when large differences are often observed between and within pipelines, with accuracy potentially remaining relatively low. For example, in each pipeline, SNV calling relies on set algorithms, which are not dramatically changed in updated versions of the software. Therefore, we do not see great leaps in accuracy for SNVs with newer versions of GATK, despite the fact that we found in the current study that at least one other pipeline (SOAP) did uniquely discover some validated SNVs that were not discovered by any other pipeline or any version of BWA-GATK tested here, and *vice versa*. We also note that GATK discovered comparatively more unique SNVs not discovered by any other pipeline. One caveat is that we processed the MiSeq data with the newest BWA-GATK pipeline, so this might favor the exome variants previously called by GATK, but it is nonetheless the case that SOAP identified variants that GATK missed in the same exome data using near-default parameters.

### Some limitations of our study

It is important to note that our study did not examine somatic mutations in tumor samples, so our conclusions and our testing pipelines focus only on germline mutations from diploid genomes. We recognize that variant calling in cancer genomes represents similar but somewhat distinct challenges, and that software tools (such as SNVMix [55]) are typically developed specifically for somatic mutations in cancer genomes. Our efforts were designed to evaluate whether or not rare variants within personal genomes can be reliably and comprehensively generated from sequencing data, with or without sequencing data from other family members. Hence, we did not evaluate pipelines that specifically employ imputation or multi-unrelated-sample variant-calling algorithms, such as Thunder [56], IMPUTE2 [57-59], or BEAGLE [60,61], or specific procedures within the software tools used that allow for calling of multiple unrelated sample (such as those available by GATK) [13]. Additionally, software tools that are only commercially available (such as CLCBio) are not evaluated in our comparative study.

## Conclusions

We have shown that there remains significant discrepancy in SNV and indel calling between many of the currently available variant-calling pipelines when applied to the same set of Illumina sequence data under near-default software parameterizations, thus demonstrating fundamental methodological variation between these commonly used bioinformatics pipelines. In spite of this methodological variation between pipelines, there exists a set of robust calls that are shared between all pipelines even under lax parameterization. We have further shown that the relatively recent CG version 2.0 WGS pipeline detects a set of exon variants that are not detected by several variant-calling pipelines with an Illumina-based exon-capture sequencing strategy, even in regions of high mappability [33]. Therefore, each single existing exon-capture, NGS platform, or variant-calling pipeline is likely to miss some true functional rare variants. Some authors have suggested using two separate sequencing platforms on the same samples [10], while others have suggested that technical replicates for exon capture may help to further improve accuracy of heterozygote variant calls [62]. With current technologies and cost considerations, exon capture and deep sequencing combined with WGS is still too expensive for most laboratories, and is therefore not likely to be a practical solution in the short term, despite providing the best combination of depth and breadth of coverage for genetic analyses of selected exonic regions. As an alternative, considering current prices, we suggest that utilizing the total list of variants derived from multiple (and orthogonal) variant-calling pipelines is a more feasible first option for reducing false negatives in a discovery setting. However, we fully acknowledge that in this scenario, the rates of false positives and false negatives are inversely and directly dependent on one another; that is decreasing the false-positive rate with filters will increase the false-negative rate, and *vice versa *[63]. We have demonstrated that studying larger multi-generational families can increase the accuracy for *de novo *variants. We also note that the standardization of indel discovery and reporting in a way that allows more accurate comparison of indels between sequencing platforms, variant-calling pipelines, and most importantly between individuals in a population is a critical step that needs to be addressed before this functionally important class of variants can be comprehensively assessed in either a research or a clinical setting.

## Abbreviations

BAM: Binary alignment map; CG: Complete Genomics; CNV: Copy number variant; eir: Equivalent indel region; Indel: Small insertion/deletion; NGS: Next-generation sequencing; SAM: sequence alignment map; SNP: Single-nucleotide polymorphism; SNV: Single-nucleotide variant; VCF: Variant call format; WGS: Whole-genome sequencing.

## Competing interests

The authors declare that they have no competing interests.

## Authors' contributions

JO, TJ, WW, LT, PB, ZW and WEJ analyzed the exome and SNP genotyping data. TJ, GS, JH and colleagues at BGI performed all exome sequencing, Sanger sequencing and Sequenom genotyping. YW performed the PCR and MiSeq validation experiments. JO analyzed the MiSeq data and Complete Genomics data, and wrote the manuscript. GJL and KW conceived of the project, supervised all data analysis, and wrote and edited the manuscript. All authors reviewed and commented on the manuscript. All authors read and approved the final manuscript.

## Description of additional data files

The following additional data are available with the online version of this paper.

Additional file 1 is a Table and 10 supplementary figures:

**Figure S1, Family pedigrees contained within the 15 sequenced exomes**. Of the fifteen exomes that were sequenced, 14 were sequenced from families chosen for future disease discovery related work. Each sequenced individual (numbered) is displayed in the context of his or her constituent family pedigree.

**Figure S2, Fraction of target capture region covered versus coverage depth for 15 exomes**. All exomes have at least 20 reads or more per base pair in > 80% or more of the 44 MB target region.

**Figure S3, Histograms of Illumina read depth at SNV coordinates**. Read depth taken from each pipeline's independently aligned BAM file at genomic coordinates of SNVs called by each of the 5 alignment and variant calling pipelines. **A) **SOAPsnp, **B) **SNVer, **C) **SAMtools, **D) **GNUMAP and **E) **GATK, respectively. Frequency of read depths for all SNVs (**A**, **B**, **C**, **D**, and **E**) as well as for SNVs having depths between 0 and 50 (**a**, **b**, **c**, **d**, and **e**) were plotted.

**Figure S4, SNV concordance measured at varying Illumina read depth threshold values**. SNV concordance for a single exome, "k8101-49685", between five alignment and variant detection pipelines: GATK, SOAPsnp, SNVer, SAMtools, and GNUMAP. Concordance between each pipeline was determined by matching the genomic coordinate as well as the base pair change and zygosity for each detected SNV. Concordance was measured at varying Illumina read depth threshold values in each independently aligned BAM file, ranging from > 0 (no threshold) to > 30 reads.

**Figure S5, Histograms of illumina read depth at genomic coordinates of the unique to Complete Genomics SNV calls**. Histograms of read depth taken from each of the five Illumina pipeline's independently aligned BAM file at genomic coordinates of SNVs that were found by Complete Genomics but not by any of the 5 Illumina pipelines: GATK, GNUMAP, SNVer, SAMtools and SOAPsnp, **A**, **B**, **C**, **D **and **E **respectively. All coordinates fell within the range of the Agilent SureSelect v.2 exons.

**Figure S6, SNV concordance for a single exome, "k8101-49685", between two sequencing pipelines: Illumina and Complete Genomics**. For the Illumina sequencing, exons were captured using the Agilent SureSelect v.2 panel of capture probes. Complete Genomics SNVs consist of a subset of all SNVs called by CG that fell within the Agilent SureSelect v.2 exons. Concordance was determined by matching the genomic coordinates, base pair composition, and zygosity status for each detected SNV. Concordance was measured between CG SNVs and **A) **the union of all SNVs called by 5 variant calling pipelines ("Illumina-data calls") and **B) **only SNVs that all 5 Illumina pipelines collectively called ("concordant Illumina-data calls").

**Figure S7, Cross-validation of illumina SNV calls using Complete Genomics SNV calls**. SNVs called by each Illumina-data pipeline were cross-validated using SNVs called by Complete Genomics, an orthogonal sequencing technology, in sample "k8101-49685". The percentage of Illumina SNVs that were validated by CG sequencing was measured for variants having varying degrees of Illumina-data pipeline concordance. The same analysis was performed for variants that were considered novel (absent in dbSNP135).

**Figure S8, Average indel concordance among 15 exomes between three indel detecting pipelines: GATK, SAMtools and SOAPindel**. Concordance was measured between raw, pre-standardized, indel calls. Indels were considered in agreement if the genomic coordinates, length and composition of indels matched between pipelines

**Figure S9, Cross-validation of illumina indel calls using Complete Genomics indel calls**. Indels called by each Illumina-data pipeline were cross-validated using indels called by Complete Genomics for sample "k8101-49685". The percentage of Illumina indels that were validated by CG sequencing was measured across varying degrees of Illumina pipeline concordance. The same analysis was done for novel indels (indels not found in dbSNP 135).

**Figure S10, A comparison between recent versions of various GATK variant calling modules**. The similarity between SNV and indel calls made between two versions of GATK, v1.5 and v2.3-9, was measured. SNV and indel calls were made using both the UnifiedGenotyper and HaplotypeCaller modules on the same k8101-49685 participant sample. Pairwise comparisons were made between the GATK UnifiedGenotyper v1.5 and each of the GATK v2.3-9 modules (the UnifiedGenotyper and HaplotypeCaller).

**Table S1, Concordance rates with common SNPs genotyped on Illumina 610K genotyping chips**.

Additional file 2 contains command-line arguments for bio-informatics pipelines and instructions for accessing data analyzed in this paper.

Additional file 3 contain data production statistics.

## Supplementary Material

Additional file 1 **Figure S1-S7**. Click here for file

Additional file 2 Command-line arguments for bio-informatics pipelines and instructions for accessing data analyzed in this paper. Click here for file

Additional file 3 **Data production statistics**. Click here for file
