# Supplementary material for: Low concordance of multiple variant-calling pipelines: practical implications for exome and genome sequencing
Source: Genome Med. 2013 Mar 27;5(3):28. doi: 10.1186/gm432 (PMC3706896; doi:10.1186/gm432)
Supplement: Additional file 1 — Figure S1-S7. [file gm432-S1.ZIP › Additional_File1/TableS1.docx]

**Table S1**.  **Concordance rates with common SNPs genotyped on Illumina 610K genotyping chips.**

| **Sample** | **Software** | **Compared Sites** | **Concordance Sites** | **Concordance** **rate** |
| --- | --- | --- | --- | --- |
| Mother-1 | SOAPsnp | 6088 | 6074 | 99.77% |
|  | GATK 1.5 | 6249 | 6224 | 99.60% |
|  | SNVer | 5723 | 5708 | 99.74% |
|  | GNUMAP | 5458 | 5434 | 99.56% |
|  | SAMTools | 5885 | 5848 | 99.37% |
| Son-1 | SOAPsnp | 6366 | 6353 | 99.80% |
|  | GATK 1.5 | 6341 | 6323 | 99.72% |
|  | SNVer | 6255 | 6239 | 99.74% |
|  | GNUMAP | 5850 | 5828 | 99.62% |
|  | SAMTools | 6383 | 6362 | 99.67% |
| Son-2 | SOAPsnp | 6412 | 6401 | 99.83% |
|  | GATK 1.5 | 6426 | 6413 | 99.80% |
|  | SNVer | 6336 | 6325 | 99.83% |
|  | GNUMAP | 5906 | 5889 | 99.71% |
|  | SAMTools | 6477 | 6450 | 99.58% |
| Father-1 | SOAPsnp | 6247 | 6238 | 99.86% |
|  | GATK 1.5 | 6304 | 6288 | 99.75% |
|  | SNVer | 6205 | 6192 | 99.79% |
|  | GNUMAP | 5805 | 5786 | 99.67% |
|  | SAMTools | 6344 | 6327 | 99.73% |

All pipelines are very good with identifying already known, common SNPs.
