# Supplementary figures and images for: Low concordance of multiple variant-calling pipelines: practical implications for exome and genome sequencing
Source: Genome Med. 2013 Mar 27;5(3):28. doi: 10.1186/gm432 (PMC3706896; doi:10.1186/gm432)

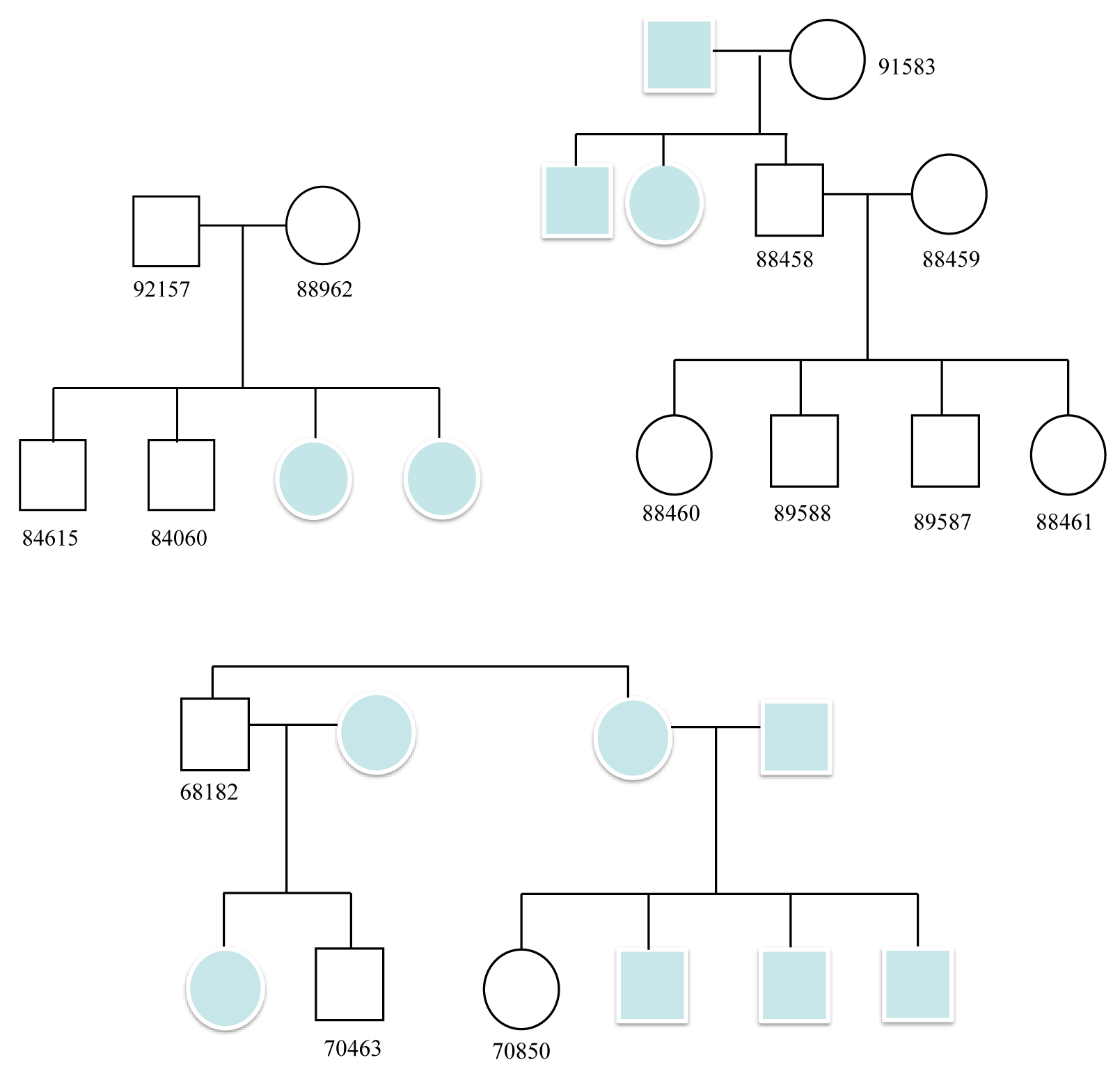

Supplement: Additional file 1 — Figure S1-S7. [file gm432-S1.ZIP › Additional_File1/FigureS1.bmp]

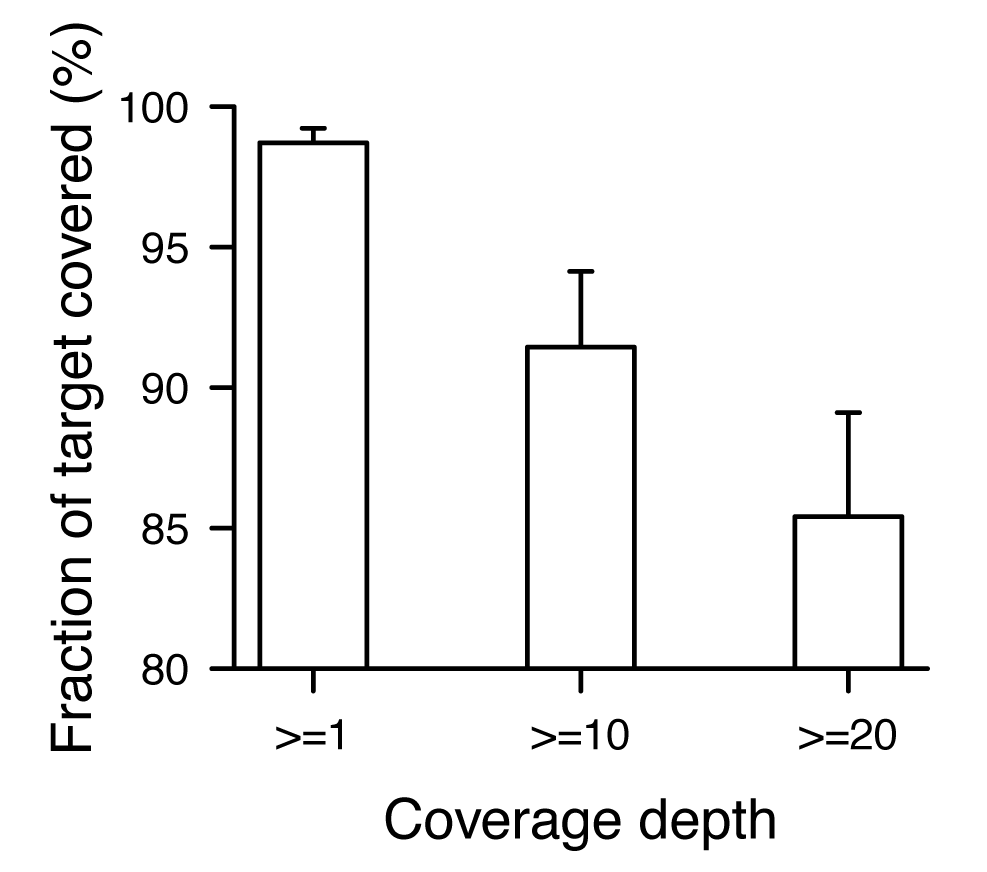

Supplement: Additional file 1 — Figure S1-S7. [file gm432-S1.ZIP › Additional_File1/FigureS2.bmp]

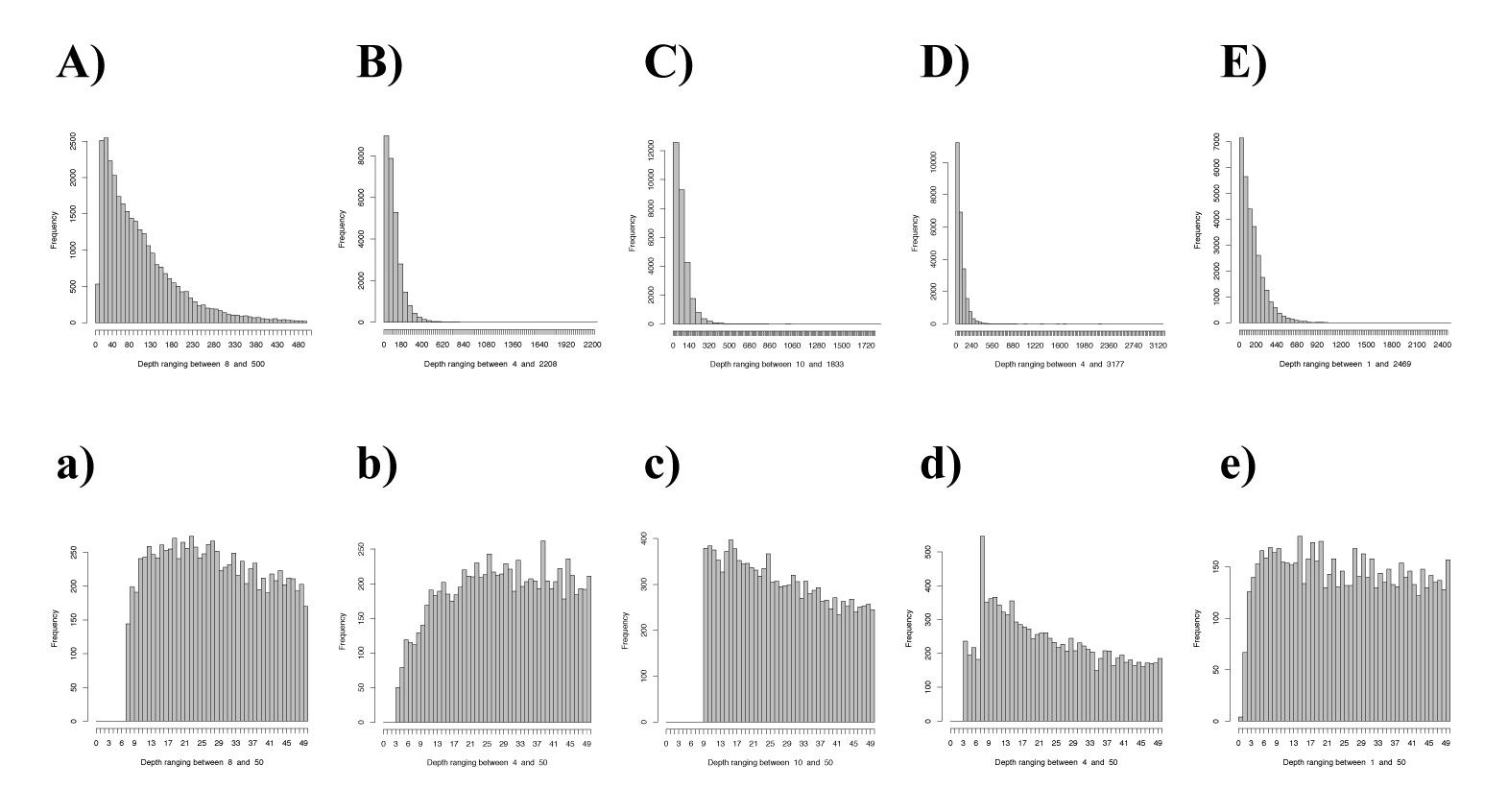

Supplement: Additional file 1 — Figure S1-S7. [file gm432-S1.ZIP › Additional_File1/FigureS3.bmp]

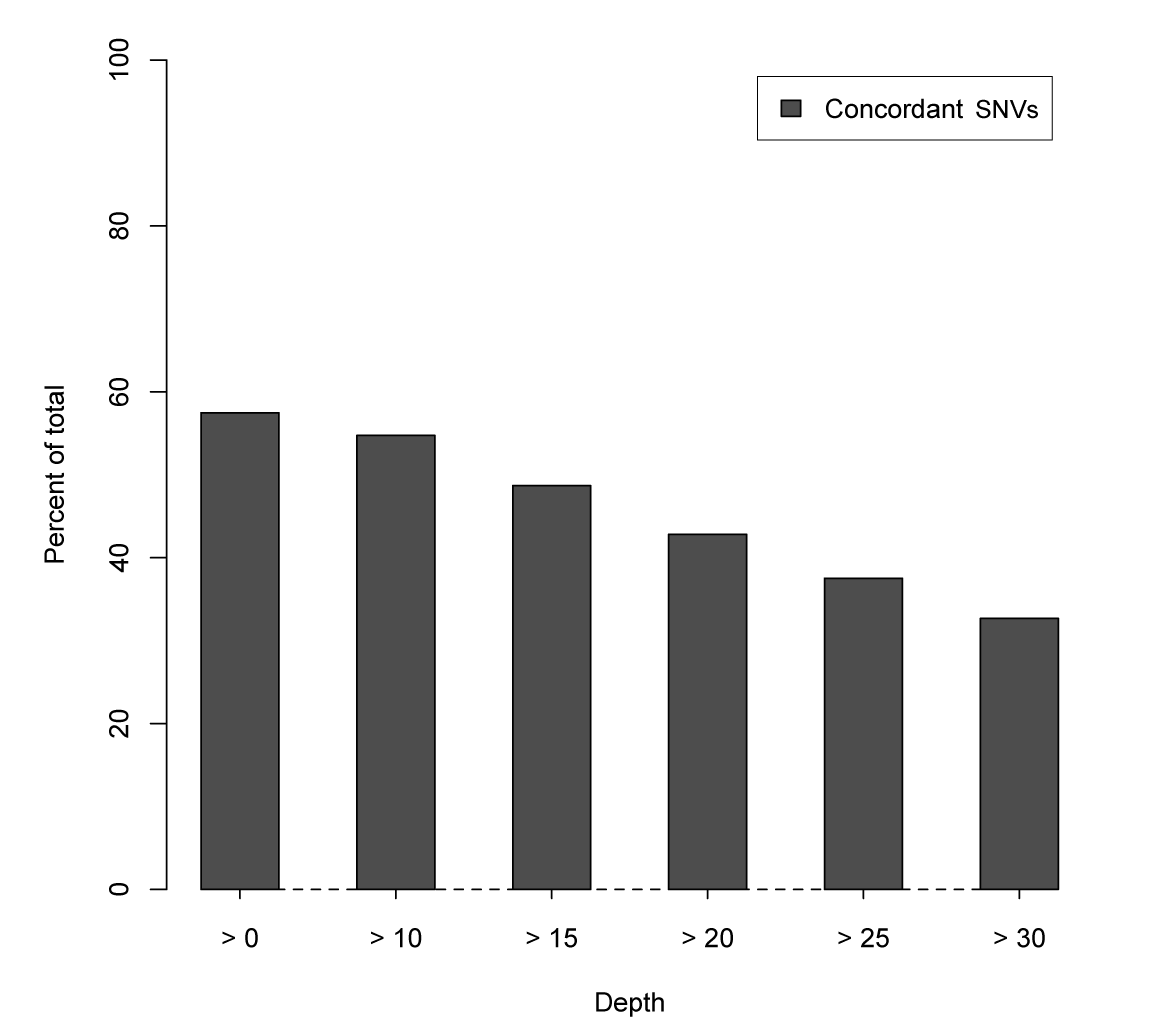

Supplement: Additional file 1 — Figure S1-S7. [file gm432-S1.ZIP › Additional_File1/FigureS4.bmp]

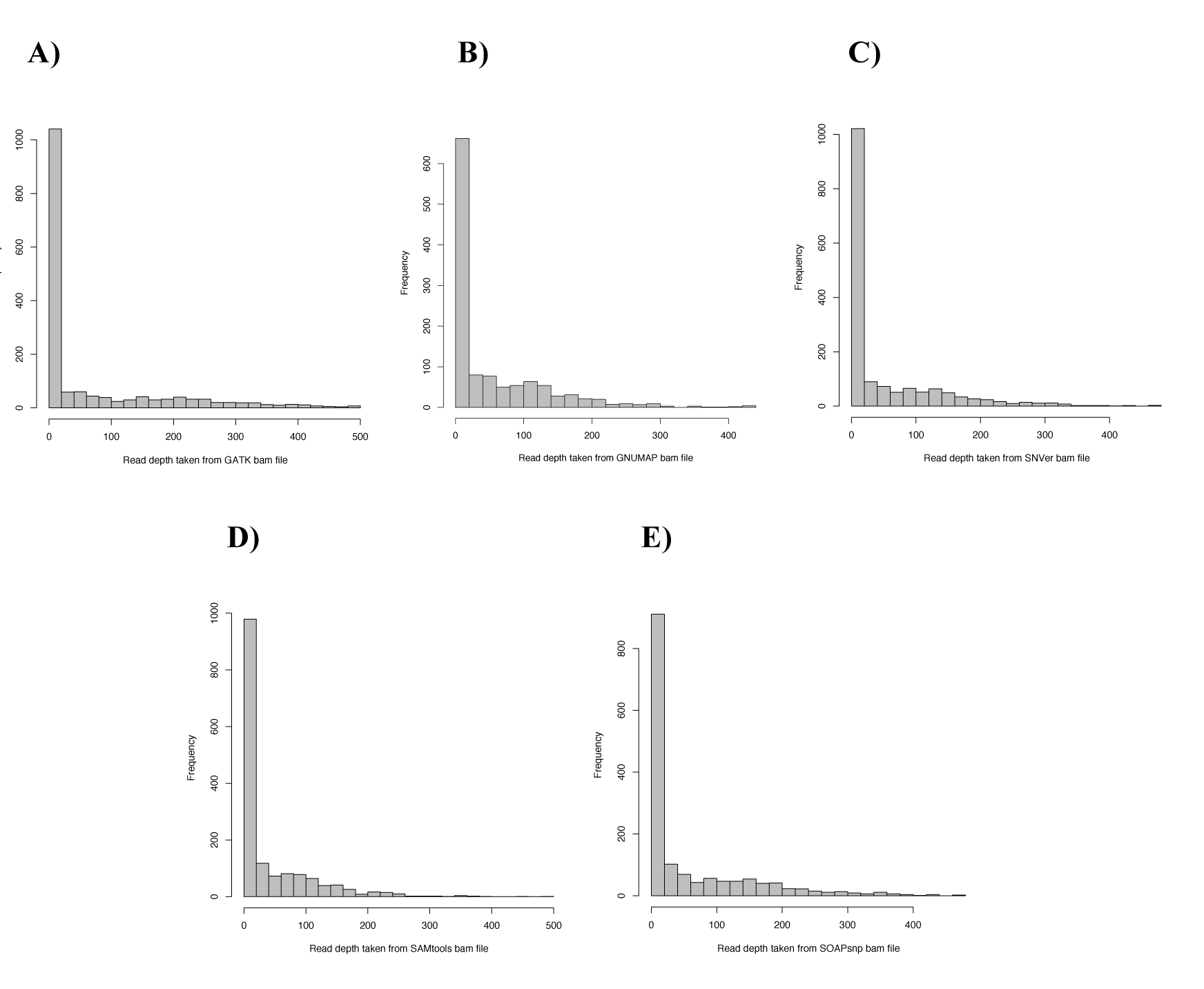

Supplement: Additional file 1 — Figure S1-S7. [file gm432-S1.ZIP › Additional_File1/FigureS5.bmp]

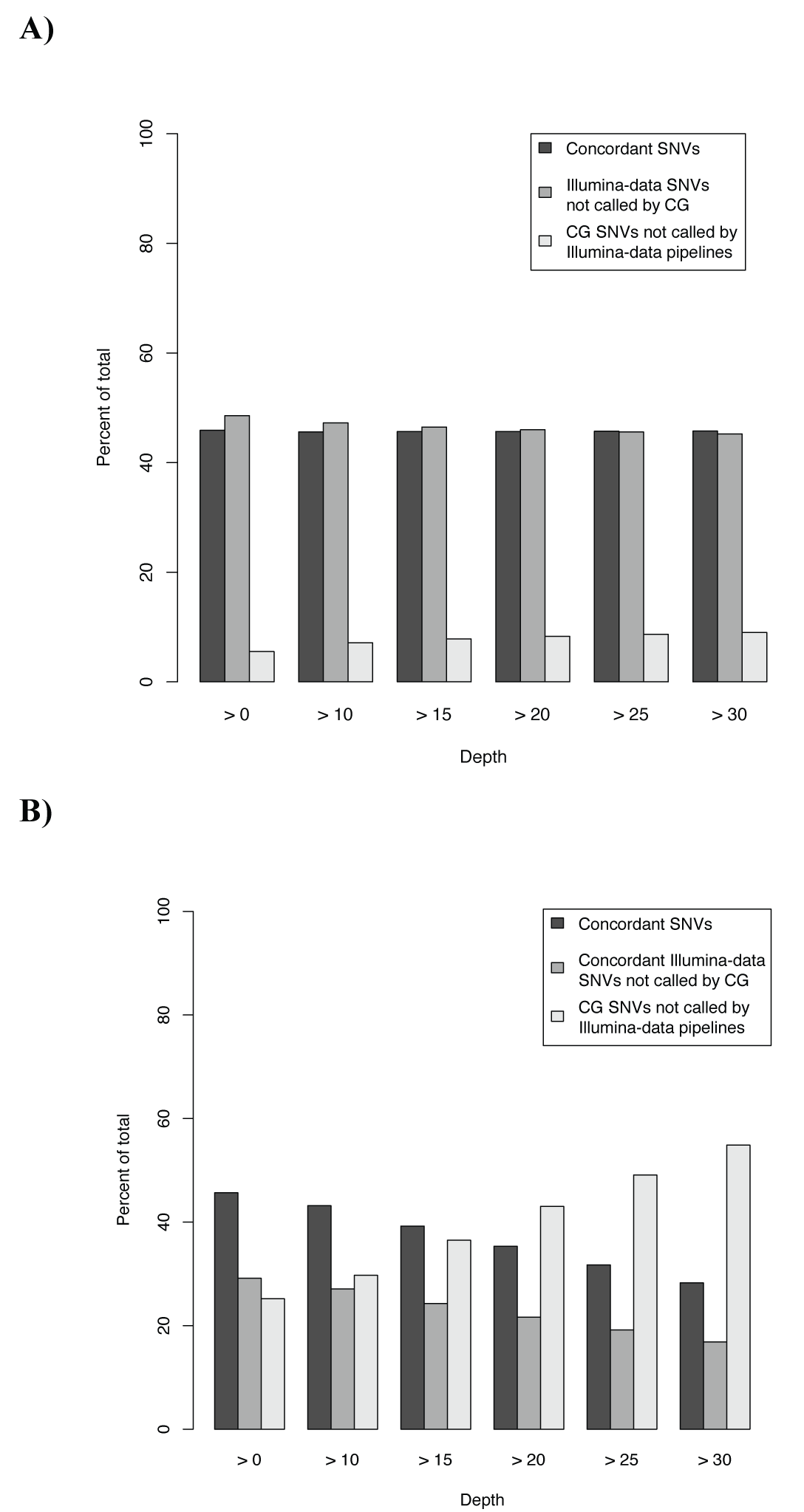

Supplement: Additional file 1 — Figure S1-S7. [file gm432-S1.ZIP › Additional_File1/FigureS6.bmp]

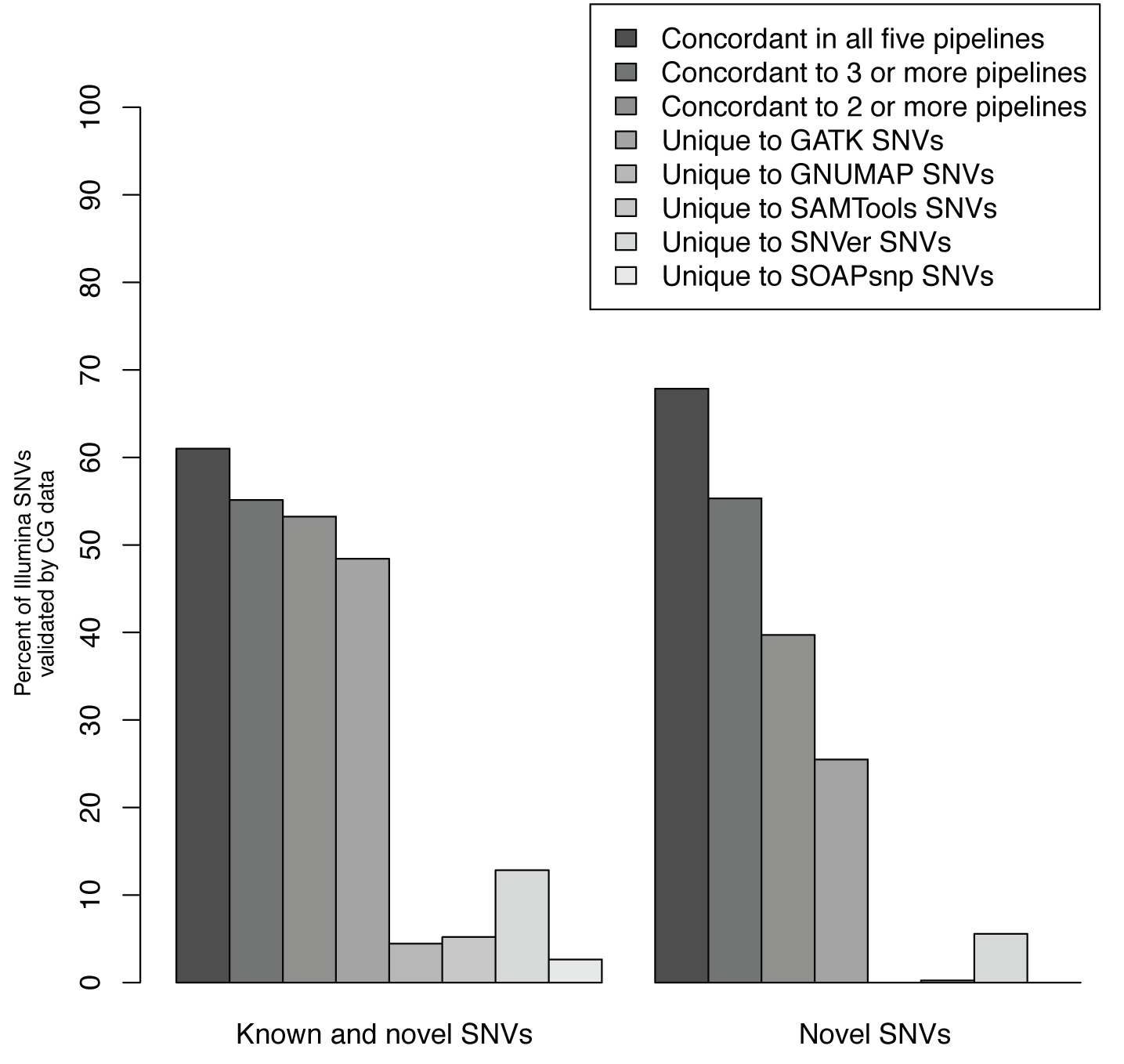

Supplement: Additional file 1 — Figure S1-S7. [file gm432-S1.ZIP › Additional_File1/FigureS7.bmp]

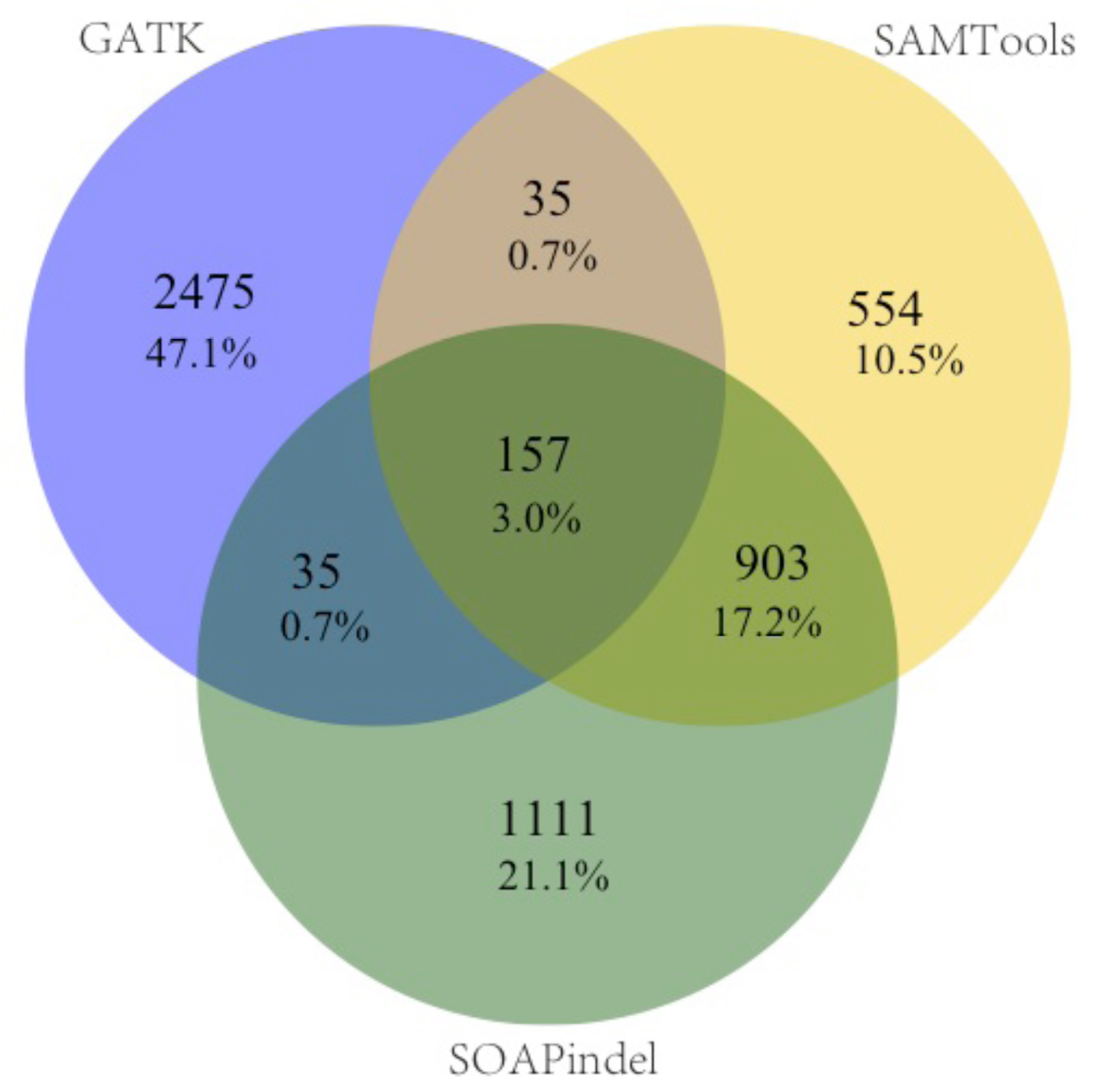

Supplement: Additional file 1 — Figure S1-S7. [file gm432-S1.ZIP › Additional_File1/FigureS8.bmp]

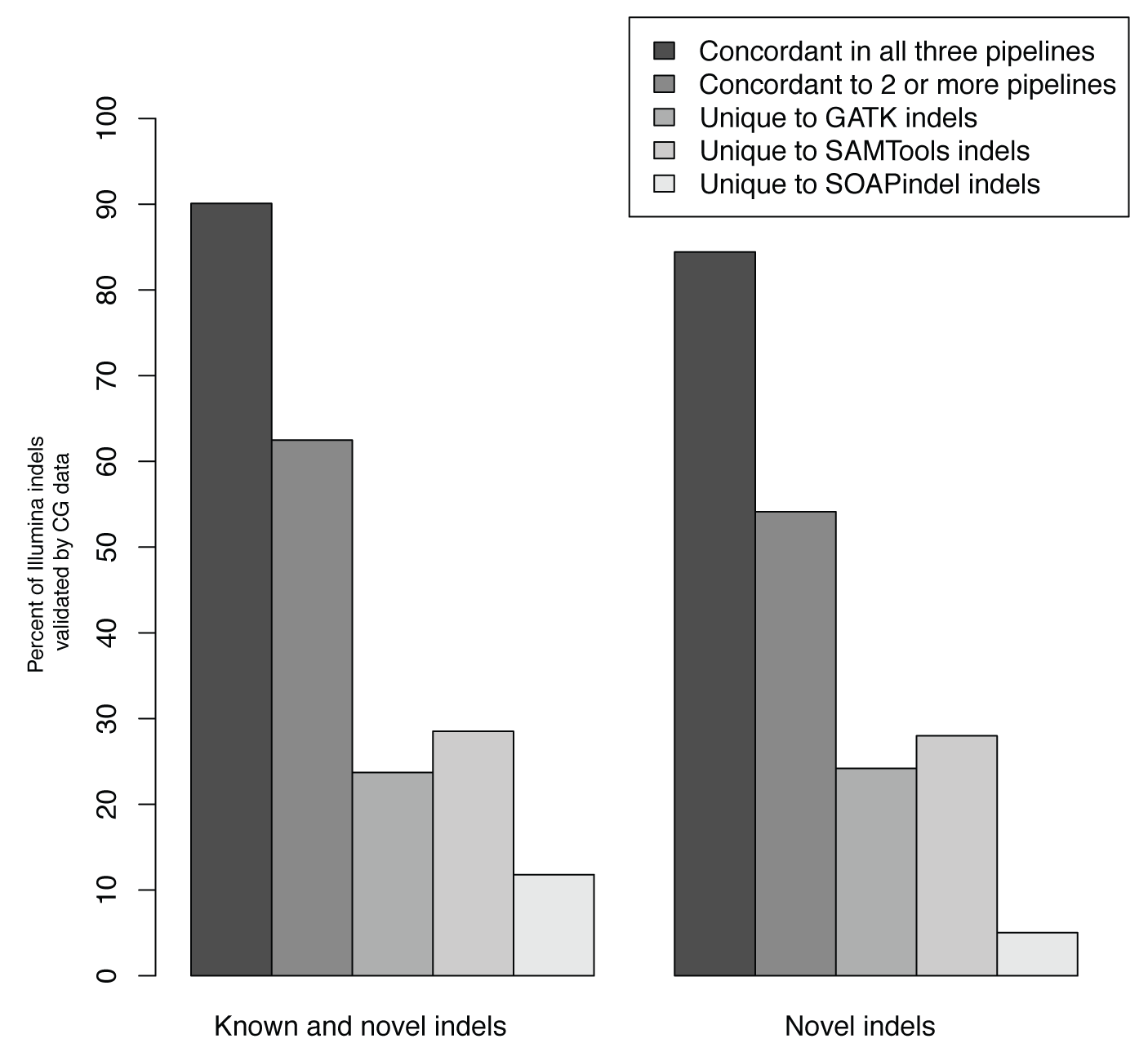

Supplement: Additional file 1 — Figure S1-S7. [file gm432-S1.ZIP › Additional_File1/FigureS9.bmp]
