# Supplementary material for: Low concordance of multiple variant-calling pipelines: practical implications for exome and genome sequencing
Source: Genome Med. 2013 Mar 27;5(3):28. doi: 10.1186/gm432 (PMC3706896; doi:10.1186/gm432)
Supplement: Additional file 2 — Command-line arguments for bio-informatics pipelines and instructions for accessing data analyzed in this paper. [file gm432-S2.DOCX]

**Supplementary document**

Table of Contents

Accessing data 2

Command-line arguments 2

GATK 2

GNUMAP 6

SAMtools 6

SNVer 7

SOAP 7

# Accessing data

All of the data have been submitted to the Sequence Read Archive under project accession SRP019719, corresponding to the 15 exomes and the single whole genome analyzed during the course of our study. Data can be acquired from the SRA website at <http://trace.ncbi.nlm.nih.gov/Traces/sra/> .

# Command-line arguments

Below are command-line arguments for various different variant discovery bioinformatics pipelines. These, as well as similar arguments, were used during the course of our study. To see the full extent of the available software options, workflows and recommended parameterization(s), visit the webpage of each respective software package:

GNUMAP : http://dna.cs.byu.edu/gnumap/

SNVer : http://snver.sourceforge.net/

SAMtools : http://samtools.sourceforge.net/

GATK : http://www.broadinstitute.org/gatk/

SOAP : http://soap.genomics.org.cn/

## GATK

$FASTQ1 = *Raw read file 1*

$FASTQ2 = *Raw read file 2*

$REFSEQ = *Reference sequence*

$KNOWNSITES =

$HAPMAP =

$ONEKG =

$GOLDENINDELS =

$AGILENTLOC =

- bwa aln $REFSEQ $FASTQ1 > 1.sai
- bwa aln $REFSEQ $FASTQ2 > 2.sai
- bwa sampe -P $genome 1.sai 2.sai $FASTQ1 $FASTQ2 > sample.sam
- samtools view -h -S -b sample.sam > sample.bam
- samtools sort sample.bam in.sort
- java -jar MarkDuplicates.jar \

INPUT=in.sort.bam \

OUTPUT=dupe.bam \

ASSUME_SORTED=true \

METRICS_FILE=dupe.metrics \

CREATE_INDEX=true \

VALIDATION_STRINGENCY=LENIENT

- java -jar GenomeAnalysisTK.jar \

-T RealignerTargetCreator \

-R $REFSEQ \

-I dupe.bam \

-o dupe.intervals \

--fix_misencoded_quality_scores

- java -jar GenomeAnalysisTK.jar \

-T IndelRealigner \

-R $REFSEQ \

-I dupe.bam \

--targetIntervals dupe.intervals \

--fix_misencoded_quality_scores \

-o realign.bam

- java -jar GenomeAnalysisTK.jar \

-T BaseRecalibrator \

-I realign.bam \

-R $REFSEQ \

-knownSites $KNOWNSITES \

-o recal_data.grp

- java -jar GenomeAnalysisTK.jar \

-T PrintReads \

-R $REFSEQ \

-I realign.bam \

-BQSR recal_data.grp \

-o realign.recal.bam

- java -jar GenomeAnalysisTK.jar \

-T UnifiedGenotyper \

-R $REFSEQ \

-I realign.recal.bam \

-glm BOTH \

-o realign.recal.vcf \

-stand_call_conf 50 \

-stand_emit_conf 10.0 \

-minIndelCnt 5 \

-indelHeterozygosity 0.0001

- java -jar GenomeAnalysisTK.jar \

-R $REFSEQ \

-T SelectVariants \

--variant realign.recal.vcf \

-o realign.recal.indel.vcf \

-selectType INDEL

- java -jar GenomeAnalysisTK.jar \

-R $REFSEQ \

-T SelectVariants \

--variant realign.recal.vcf \

-o realign.recal.snp.vcf \

-selectType SNP

- java -jar GenomeAnalysisTK.jar \

-T VariantRecalibrator \

-R $REFSEQ \

-input realign.recal.snp.vcf \

-recalFile snp.output.recal \

-tranchesFile snp.output.tranches \

--maxGaussians 4 \

-resource:hapmap,known=false,training=true,truth=true,prior=15.0 $HAPMAP \

-resource:omni,known=false,training=true,truth=false,prior=12.0 $ONEKG \

-resource:dbsnp,known=true,training=false,truth=false,prior=6.0 $KNOWNSITES \

-an QD -an HaplotypeScore -an MQRankSum -an ReadPosRankSum -an FS -an MQ \

-mode SNP \

- java -jar GenomeAnalysisTK.jar \

-T ApplyRecalibration \

-R $REFSEQ \

-input realign.recal.snp.vcf \

--ts_filter_level 99.0 \

-tranchesFile snp.output.tranches \

-recalFile snp.output.recal \

-mode SNP \

-o realign.recal.filtered.snp.vcf

- java -jar GenomeAnalysisTK.jar \

-R $REFSEQ \

-T SelectVariants \

--variant realign.recal.filtered.snp.vcf \

-o output.vcf \

-L $AGILENTLOC

- java -jar GenomeAnalysisTK.jar \

-R $REFSEQ \

-T SelectVariants \

--variant output.vcf \

-o output.PASS.vcf \

-ef

- java -jar GenomeAnalysisTK.jar \

-T VariantFiltration \

-R $REFSEQ \

-o realign.recal.filter.indel.vcf \

--variant realign.recal.indel.vcf \

--filterExpression "QD < 2.0 || FS > 200.0" \

--filterName "FSFilter"

- java -jar GenomeAnalysisTK.jar \

-R $REFSEQ \

-T SelectVariants \

--variant realign.recal.filter.indel.vcf \

-o output.indel.vcf \

-L $AGILENTLOC

- java -jar GenomeAnalysisTK.jar \

-R $REFSEQ \

-T SelectVariants \

--variant output.indel.vcf \

-o output.indel.PASS.vcf \

-ef

- java -jar GenomeAnalysisTK.jar \

-T HaplotypeCaller \

-R $REFSEQ \

-I realign.recal.bam \

--dbsnp $KNOWNSITES \

-stand_call_conf 50.0 \

-stand_emit_conf 10.0 \

-o haplotype.realign.recal.vcf

- java -jar GenomeAnalysisTK.jar \

-T VariantRecalibrator \

-R $REFSEQ \

-input haplotype.realign.recal.vcf \

-recalFile hapmap.output.recal \

-tranchesFile hapmap.output.tranches \

--maxGaussians 6 \

-resource:hapmap,known=false,training=true,truth=true,prior=15.0 $HAPMAP \

-resource:omni,known=false,training=true,truth=false,prior=12.0 $ONEKG \

-resource:mills,known=true,training=true,truth=true,prior=12.0 $GOLDENINDELS \

-resource:dbsnp,known=true,training=false,truth=false,prior=6.0 $KNOWNSITES \

-an QD -an MQRankSum -an ReadPosRankSum -an FS -an MQ -an ClippingRankSum \

-mode BOTH

- java -jar GenomeAnalysisTK.jar \

-T ApplyRecalibration \

-R $REFSEQ \

-input haplotype.realign.recal.vcf \

--ts_filter_level 97.0 \

-tranchesFile hapmap.output.tranches \

-recalFile hapmap.output.recal \

-mode BOTH \

-o haplotype.realign.recal.filtered.vcf

- java -jar GenomeAnalysisTK.jar \

-R $REFSEQ \

-T SelectVariants \

--variant haplotype.realign.recal.filtered.vcf \

-o haplotype.realign.recal.filtered.agilent.vcf \

-L $AGILENTLOC

- java -jar GenomeAnalysisTK.jar \

-R $REFSEQ \

-T SelectVariants \

--variant haplotype.realign.recal.filtered.agilent.vcf \

-o haplotype.realign.recal.filtered.agilent.indel.vcf \

-selectType INDEL

- java -jar GenomeAnalysisTK.jar \

-R $REFSEQ \

-T SelectVariants \

--variant haplotype.realign.recal.filtered.agilent.vcf \

-o haplotype.realign.recal.filtered.agilent.snp.vcf \

-selectType SNP

- java -jar GenomeAnalysisTK.jar \

-R $REFSEQ \

-T SelectVariants \

--variant haplotype.realign.recal.filtered.agilent.indel.vcf \

-o haplotype.realign.recal.filtered.agilent.indel.PASS.vcf \

-ef

- java -jar GenomeAnalysisTK.jar \

-T VariantFiltration \

-R $REFSEQ \

-o haplotype.realign.recal.filtered.agilent.indel.PASS.strand.vcf \

--variant haplotype.realign.recal.filtered.agilent.indel.vcf \

--filterExpression "QD < 2.0 || FS > 200.0" \

--filterName "FSFilter"

- java -jar GenomeAnalysisTK.jar \

-R $REFSEQ \

-T SelectVariants \

--variant haplotype.realign.recal.filtered.agilent.snp.vcf \

-o haplotype.realign.recal.filtered.agilent.snp.PASS.vcf \

-ef

## GNUMAP

$REFSEQ = *Reference sequence*

$SPECIMEN = *Exome-specific identifier*

$SEQFILES = *Raw read files specific to* $SPECIMEN

- gnumap -g $REFSEQ -o $SPECIMEN.out -v 1 -m 12 -j 10 -h 100 -a .95 -p -c 8 --MPI_largemem \"$(echo $SEQFILES | sed -e 's/ /,/g')\"

$SAMFILE = *Resultant SAM file*

- grep "XA:f:2[67]" $SAMFILE

$INPUT = *Sorted, concatenated SAM file*

- sam2sgr -g $REFSEQ -o $OUTPUT -v 1 -c 12 --snp $INPUT

## SAMtools

$REFSEQ = *Reference sequence*

$INPUTBAM = *Input BAM file*

$OUT = *Output vcf*

- samtools mpileup -u -f $REFSEQ $INPUTBAM | bcftools view -vcg - > $OUT

## SNVer

$REFSEQ = *Reference sequence*

$READ1 = *Raw read file 1*

$READ2 = *Raw read file 2*

$SEQID = *Sequence ID*

$SEQINDENT = *Sequence Identifier*

- bwa aln -I $REFSEQ $READ2 > 2.sai
- bwa aln -I $REFSEQ $READ1 > 1.sai
- bwa sampe -r '@RG\tID:$SEQID\tSM:$SEQINDENT\tPL:Illumina' $REFSEQ 1.sai 2.sai $READ1 $READ2 > $SEQINDENT.sam
- samtools view -h -u -S -F 12 -f 2 $SEQINDENT.sam | samtools sort - $SEQINDENT.sam.sorted
- java -jar MarkDuplicates.jar ASSUME_SORTED=true REMOVE_DUPLICATES=true I=$SEQINDENT.sam.sorted.bam O=$SEQINDENT.sam.sorted.dedup.bam M=$SEQINDENT.sam.sorted.metrics VALIDATION_STRINGENCY=SILENT
- java -jar SNVerIndividual.jar -i $SEQINDENT.sam.sorted.dedup.bam –r $REFSEQ -l target.bed

## SOAP

$REFSEQ = *Reference sequence*

$REFSEQINDEX = *Reference sequence index*

$READ1 = *Raw read file 1*

$READ2 = *Raw read file 2*

$LIST = *Mapping list*

$DIR = *Working directory*

- soap -D $REFSEQINDEX -a $READ1 -b $READ2 -o alignment.output -2 unpaired.output -v 5
- msort -k 8,n9 alignment.output > alignment.output.sort
- soapsnp -i alignment.output.sort -d $REFSEQ -o chr.consensus -r 0.00005 -e 0.0001 -t -u -L 150 -M chr.mat
- perl indel_detection.ibam.pl -wd $DIR -st samtools $LIST $REFSEQ
